# The effect of dance movement therapy on traumatized refugee women – a clinical controlled trial

**DOI:** 10.1186/s12906-026-05299-1

**Published:** 2026-09-25

**Authors:** Sabine C. Koch, Zahra Nazemi, Christina Arnaud, Kayleigh Cook, Sonja J. Steltmann, Crystal Tomaszewski

**Affiliations:** 1 Research Institute of Creative Arts Therapies (RIArT), Alanus University, Bonn, Germany; 2 School of Health, Education and Social Sciences, SRH University, Heidelberg, Germany; 3 Faculty of Fine Arts, CATRU, University of Melbourne, Melbourne, Australia; 4 Department of Psychology, University of Heidelberg, Heidelberg, Germany; 5 Department of Psychology, University of Mainz, Mainz, Germany; 6 Laboratoire de Psychologie (UR 3188), Université Marie Et Louis Pasteur, Besançon, 25000 France

**Keywords:** Dance Movement Therapy, Refugees, Displacement, Clinical Controlled Trial, Trauma, Stress, Depression, Anxiety, RHS-15, Cultural Adaptation

## Abstract

**Background:**

Women worldwide are fleeing war, persecution and violence. Typically, they experienced multiple traumata in the home country, on the way, and in the host country. In Germany, less than 10% of the refugees in need receive mental health treatment. Dance movement therapy (DMT) was tested as a treatment option for traumatized refugee women regarding its potential to reduce symptoms of trauma, stress, depression and anxiety.

**Methods:**

In a controlled within-group design, *N* = 24 traumatized refugee women from six countries, dwelling in communities of Southern Germany, were tested at baseline (t1), pretest (t2), and posttest (t3), with an exploratory follow-up (*N* = 10) on symptoms of trauma, stress, depression and anxiety. We employed the RHS-15 as the primary outcome measure, and the DASS21 and PCL-S as secondary measures. Scales were administered in an interview format in the participants’ native or a well-spoken language. After an initial 8-week waiting period, the intervention—integrating participants’ emotionally meaningful music and their own dance improvisations—was conducted in 8 weekly group sessions of 1.5 h. In the study period, no woman had any other psychotherapy. Results were analyzed with Greebhouse-Geisser corrected F-tests and with t-tests for related samples.

**Results:**

Results suggest that (a) the trauma symptoms of all women were clinically relevant at baseline, that (b) all symptoms remained unchanged after the waiting period, and that (c) symptoms of trauma, stress, depression, and anxiety dropped significantly after the DMT intervention; in the case of trauma (PCL-S) so markedly that they fell under the clinical cutoff value. The exploratory follow-up suggests that effects carry on after eight weeks, but not after one year. Limitations of the study are the use of a within-group design, and a relatively small, yet fully powered, sample.

**Conclusions:**

The refugee women in this study experienced a pronounced symptom reduction regarding trauma, stress, depression, and anxiety after an 8-week DMT intervention. Based on this preliminary evidence, DMT seems to be a promising low-threshold and cost-effective therapy approach that can improve the mental health and wellbeing of refugee women of diverse cultural backgrounds suffering from PTSD symptoms. The lack of high-quality studies with proper control conditions and follow-ups points to the need for future research with more rigorous methodologies.

**Trial registration:**

OSF clinical trial registration (retrospectively registered at Center for Open Science on 27/03/2023). https://doi.org/10.17605/OSF.IO/EG7SD.

**Supplementary Information:**

The online version contains supplementary material available at https://doi.org/10.1186/s12906-026-05299-1.

## Introduction

The global refugee crisis has reached unprecedented levels, displacing millions due to war, civil war, persecution, and natural disasters. From 2012 to 2022, the global refugee population has more than doubled, with numbers increasing from 48.2 million to 100 million in just that one decade; presently, there are approximately 122 million displaced persons world-wide [1]. Women often carry the main burden of migration in terms of care for vulnerable others, and regarding mental health vulnerability [2].

### Refugee mental health

The WHO defines mental health (MH) as “a state of wellbeing that enables people to cope with the stresses of life, to realize their abilities, to learn well and work well, and to contribute to their communities” [3]. Although migration may in some cases bring benefits for mental health and well-being of refugees, many are subject to an increased risk of experiencing adverse mental health conditions [2].

Mental health disorders are prevalent among refugees, with high rates of posttraumatic stress disorder (PTSD), depression, and anxiety reported across various studies [4, 5]. PTSD can occur following exposure to potentially traumatic events (e.g., war, suffering or witnessing violence) and is characterized by symptoms of re-experiencing, avoidance, and hyperarousal [6]. The traumatic experiences endured by refugees, coupled with the stressors of resettlement, create a complex psychological situation that requires comprehensive and culturally sensitive interventions.

WHO (2023) reports that among migrant and refugee children in Europe [7, 8], girls displayed higher levels of depression and anxiety, boys showed higher levels of behavioral disorders, while PTSD prevalence was independent of gender, mirroring trends in the general population. Female gender was a risk factor for the prevalence of depression and anxiety in unaccompanied minors [9]. Among adult refugees, women were more likely than men to experience PTSD after witnessing the same traumatic event [2, 10]. Women often have additional risk factors such as low literacy, high social isolation, marginalization, and specific flight reasons such as child marriage, trafficking and continued sexual and gender-based violence [2, 10–12]. Conversely, male refugees had a higher risk of developing a psychotic disorder [2].

### Refugees as an underserved population in MH treatment

Compared to acute physical or severe chronic diseases, mental health is a largely underserved area for refugees [2]. Globally, MH services for refugees are characterized by missing access and inadequate service provision [13–16]. Barriers to MH service access for refugees include lack of cultural awareness in the health systems, lack of culturally diverse health professionals, limited knowledge about refugees and limited access to interpreters [17].

Such barriers can emerge right from the point of requesting an appointment. In Germany, a field experiment conducted on 6,800 randomly selected practices of registered doctors (general medicine, dermatology, pediatrics and radiology) and psychotherapists demonstrated that the probability of receiving a positive response to the request with a name that was registered in Nigeria or Turkey was six to eight percentage points lower than with a name common in Germany. The greatest difference was found among psychotherapists [18–20].

In Germany, in the first 36 months after arrival, refugees are only entitled to medical treatment for acute illnesses and pain. After that period, it is often difficult to provide appropriate medical and therapeutic care, because interpreters are not funded, and healthcare staff lack resources and cultural skills or training in the contexts of flight and human rights violations [19]. Other post-migration stressors often contribute to increasingly poor mental health outcomes in the German context [21], racism being one of them [20]. To counteract this care deficit, forty-eight German Psychosocial Centers (PSC), organized under the umbrella of the Federal Association of Psychosocial Centers for Refugees and Torture Victims (BAfF), offer specialized multi-professional services. The PSCs provide psychological, therapeutic, social work and legal counseling services for those who have experienced flight and torture. Interpreters are available [19]. In 2022, the PSCs provided care for 25,861 clients. Despite the continuous increase in the number of clients, the PSCs and their cooperation partners were only able to cover 3.1 percent of the potential need for mental health care in 2022 [19].

### Dance Movement Therapy (DMT) as an arts-based treatment option

The arts are gaining momentum as a treatment option for underserved populations [22]. The low-threshold, easy-to-access creative arts therapies modalities, such as art therapy, music therapy, dance movement therapy or drama therapy, provide pathways to treatment that are resource-oriented and playful, and often lead around the stigma of MH treatment and psychotherapy that is still prevalent in many societies. In creative arts therapies (CATs) [23], the aesthetic experience is considered an important therapeutic factor and potential predictor of health, highlighting the significance of beauty in the therapeutic process [24, 25], and its importance in promoting psychological well-being. All trauma has components that occur on the body level. Dance Movement Therapy (DMT), as one of the arts modalities, is a promising therapeutic approach to address the MH needs of refugees by working with the body. According to the American Dance Therapy Association [26], DMT is defined as the psychotherapeutic use of movement to promote emotional, social, cognitive, and physical integration of the individual. By engaging the body in the therapeutic process, DMT helps individuals express and process emotions without or with less words, build resilience, and foster a sense of community and belonging [26]. Moreover, DMT is increasingly building a strong evidence-base in mental and physical health [27–31].

### An overview of DMT for refugees

For an overview of the work done in the field of DMT for refugee populations, we reviewed four major recent systematic reviews (SRs) [32–35]. The systematic reviews in the area of DMT for trauma teach us that there are no randomized controlled trials (RCTs) on DMT for refugees, it is basically only qualitative studies [34]. Four studies on DMT for refugees out of 15 studies on DMT for trauma were found in the systematic review of Tomaszewski et al. (2023), and five studies on DMT for refugees out of 16 on DMT for trauma were found in the systematic review by Liang and Bryant (2024). Another systematic review [35, 36] on body and movement therapies for trauma by van de Kamp et al. (2019/2023) was focused on body psychotherapy (BP) and on trauma in mostly military contexts, with two of the 29 studies on refugees [37, 38], but none on DMT for refugees. Their meta-analysis suggests a mean *Hedges’g* effect size of 0.50, 95% *CI* [0.22, 0.79] of body psychotherapy for decreasing PTSD symptoms, with a very high heterogeneity, *I*2 = 89%. Meta-analyses of secondary outcomes resulted in *Hedges’g* effect sizes of 0.37 for depressive symptoms; 0.62 for sleep quality, and − 0.10 for interoceptive awareness. Only one study had a low risk of bias. The work with traumatized military is gaining momentum in CATs, and beneficial effects of arts-based approaches are increasingly reported [39, 40].

From the nine DMT for refugee studies reported in the SRs of Liang and Bryant (2023) and Tomaszewski et al. (2023), two studies overlap [41, 42], and one is another review [43] reporting 11 qualitative and case studies on body- and arts-based approaches between 2010 and 2020, so that six primary studies on DMT for refugees remain. All of them were qualitative or mixed methods studies [41, 42, 44–47], with Ley (2017) being a sport and exercise therapy study, but using DMT principles, which is why we kept it in. The additional review of Schaeffer and Cornelius-White (2021) contributes, five studies on CATs next to the body therapy papers [48], three case studies on drama therapy for refugees [49–51], and two more studies on DMT for refugees [52, 53]. Schaeffer and Cornelius-White (2021) emphasize that DMT improves physical health, emotional experience and bodily awareness in refugees via the creation of a safe space, ritual, symbols and metaphors in dance. DMT enhanced embodiment, freedom of expression and empowerment, with participants feeling an increase in their presence and interpersonal skills [32]. In sum, we found eight empirical studies on DMT for refugees from systematic reviews, and three from hand search, all of them qualitative and without a control group. Table 1 provides an overview of the results.

**Table 1:** N=11 ‘DMT for refugees’-studies detected by systematic search and hand search; all facilitators (except for in the Ley-study, which still also employed DMT methods) were trained dance movement therapists; DMT=dance movement therapy, min=minutes, y=year.

#	Author, year	N, characteristics	Main finding
1	Callaghan, 1998 (hand search)	DMT groups (no numbers), male refugees from African and Asian countries in U.K	Improved self-esteem and confidence
2	Gray, 2001 [44]	Group (diverse refugee groups) and individual (N = 1); no numbers	Improved posture & confidence; after torture an adult survivor’s sense of wholeness, interaction skills and relationship capacity improved
3	Koch & Weidinger v. d. Recke, 2009 [41]	N = 9 (group), N = 1 (indiv.); nine Albanian women from Kosovo suffering from PTSD; one woman from Togo	Group: DMT emotionally engaged participants and reduced feelings of shame and guilt, cultural verbal and non-verbal support was important, re-integration of the fragmented and negatively cathected body image was facilitated, inner stability grew, presence of others helped to prevent dissociation & re-traumatization; individual: increase in self-confidence
4	Verrault, 2017 [42]	N = 8, eight female asylum seekers and refugees, from 19 to 50 y. with traumatic complaints, depressive and somatic symptoms. Intense program of four weekly DMT sessions of 75 min for one month (a drama therapist co-facilitated)	DMT ameliorated body awareness, increased the feeling of safety and religious, spiritual, psychological, and cultural resources, and provided group support
5	Ley et al., 2017 [45]	N = 4; four refugees, war- and torture survivors, with post-traumatic stress disorders, depression, anxiety disorders a.o. physical, psychosomatic, and psychosocial symptoms; twice a week for 3 months (2 physical education trainers + a trauma expert following DMT methods)	Results suggest that achieving flow is important for dance therapy to be effective. It can improve the affective state and create a new sense of safety & confidence
6/7	Harris, 2007a, b (hand search) [54]	N = 12; 16 sessions; well-described intervention, suggests therapeutic factors	Self-reported ratings for symptoms of aggressive behavior, depression, anxiety, intrusive recollections and elevated arousal decreased from intake to reassessment
8	Harris, 2019 [46]	DMT group with former child soldiers from Africa	Suggests that movement affords an enhanced sense of safety, thereby allowing survivors to move from a passive to an active stance
9	Rapsari & Hill, 2019 [53]	N = 3; case study with two men and one woman from Burma in the US (37-45y). Therapists incorporated group members’ cultural and religious identities in their interventions, activating their existing internal resources	Embodiment enabled participants to activate internal resources and personal resilience through their bodies. Intentional, imaginative embodiment of metaphors or roles helped participants to process and re-write their journeys including traumatic experiences. DMT was able to bridge cultural and language gaps between participants
10	García-Medrano & Panhofer, 2020 [52]	N = 51, in a study with three groups of migrants in Glasgow (45 women, 6 men) conducted ‘spontaneous movement groups’ based on clinician’s DMT, Gestalt, and BP training N = 1, case study included. Focused on embodiment as an active factor evoking emotions and memories held in the body, and the body’s ability to express internal, preverbal or traumatic states in conjunction with metaphor	DMT was particularly suitable for cross-cultural treatment and connection among culturally diverse group members. Gradual development of one participant’s confidence reflected in changes in her posture, movement, vocal quality, and dress over time. Results suggest increased body awareness, resilience, confidence and interpersonal trust, a beneficial sense of social belonging, and decreased symptoms of stress, anxiety & depression
11	Dieterich-Hartwell et al., 2021 [47]	N = 5 (individual), N = 7 (group); DMT sessions of 60 min for refugees with a trauma history, who had lived in the US for at most five years. assessing general resources regarding body/movement; 3–5 meetings (60 min) to explore and develop expressive movement phrases and movement experience	DMT allowed participants to experience expressive movements, bear active movement factors, and extended resources (cultural, environmental, family)

In sum, all eleven studies report positive outcomes, such as improved feeling of safety, body awareness, confidence, self-esteem, increased active stance, and no aversive events (see Table 1). DMT methods and approaches for trauma treatment have been vastly described [54–57]. In her trauma model, Dieterich-Hartwell (2017) reports that trauma survivors often describe a disconnection from and lack of safety within their bodies, along with an inability to identify the meaning of physical sensations and muscle activation. These challenges are particularly pronounced in individuals with severe post-traumatic stress disorder (PTSD) symptoms, including flashbacks, dissociative episodes, hypervigilance, and emotional numbness. Such profound disconnection and distress underscore the need for therapeutic interventions that can help survivors to re-establish a sense of safety [58] in the body.

### Our study

From the four reviews including the previously conducted DMT for refugee studies, all qualitative in methodology, it becomes clear that quantitative data and controlled studies are missing. Our study therefore set out to fill this gap and contribute to the question of efficacy of DMT on the highest evidence-level feasible, adding a controlled study. In today’s health care systems, treatment options need to be demonstrating effects on the highest possible evidence level to enter medical guidelines and to be considered for funding. Given the unavailability of MH treatment for most traumatized refugees in Germany, we aimed to contribute data for improving the situation of this most vulnerable group of persons. We investigated whether DMT is an effective and culturally acceptable treatment option for addressing trauma and adjacent MH symptoms in refugee women in Germany.

Our research question was “Can dance movement therapy (DMT) support traumatized refugee women by decreasing symptoms of trauma, stress, depression and anxiety?” We tested the hypothesis *H0: DMT cannot significantly decrease symptoms of trauma, stress, depression and anxiety* versus the hypothesis *H1: DMT can reduce symptoms of trauma, stress, depression and anxiety* (employing RHS-15, PCL-S, and DASS21).

## Methods

The general aim of the study was to test whether DMT can decrease symptoms of trauma, stress, depression, and anxiety in a sample of traumatized refugee women in Southern Germany.

### Sample and design

The design was a one-factorial within-group comparison of *N* = 24 women, comparing a waiting control period with an intervention period, and an exploratory follow-up. Refugee women had a mean age of *M* = 39,08 (*SD* = 12,47, *range* = 20–65), and came from Ukraine, Afghanistan, Turkey, Nigeria, Cameroon with self-defined trauma, recruited through social workers, self-support groups, and the BIOS Psychosocial Center for Refugees & Torture Victims Nordbaden (PCS Nordbaden).^1^ They received eight sessions of DMT (once a week for 1.5 h) in three successive factual therapy groups of 7(4), 11(10) and 12(10) participants. From initially 30 women with the intention-to-treatment, 24 remained for analysis (drop-out reasons are listed below). For the time of the study (January 2023 till July 2024), neither woman had any other psychotherapy. At the end of the 8th session after the t3 interview, the woman received a small gift for their participation in the study (earrings, a hand flatterer, and sweets; monetary value < 10€).

For the focus group interview *N* = 10 women of group 3 participated after their final session. For the follow-up in July 2024, we reinvited all 24 women to a final questionnaire interview and DMT session (four were no longer reachable). *N* = 10 from all three therapy groups participated; five from the first year and five from the second year of the study. While our methodological choice would have been to conduct an RCT, the appearance of only seven women in the first round made it impossible to create two groups. In the end, our statistical consultant recommended within group comparisons, for which the sample was fully powered assuming medium effect sizes.

#### Recruitment

Participants were recruited via flyers, emails, phone calls, and referrals from social workers (particularly through PSC Nordbaden, city administration and NGOs), and a Ukrainian self-help WhatsApp group, which brought in most of the participants. Flyers announced “Dance movement therapy for traumatized refugee women” with a brief incentive text. We were aware that this would attract mostly self-defined traumatized women (or ‘traumatized’ as defined by their social workers), and that we would have to see whether their scores on standardized measures would indicate clinically relevant trauma symptoms.

#### Dropouts

Initially, 30 women were recruited, 24 remained for analysis. Dropout reasons included returning to their home country (2 returning to their University studies in Ukraine), somatic complaints (1), loss of contact after the initial interview (1). Two women participated in less than four sessions: only women who participated in at least 50% of the intervention sessions were included in the analysis.

### Procedure

The women took part in an 8-session DMT group intervention of 1.5 h each week at a central dance studio in Heidelberg, Germany. Therapists, co-therapists and helpers from SRH DMT Master program, researchers from Alanus University, and the collaborating University of Heidelberg and University of Bourgogne in Dijon, were part of the study. We conducted three factual therapy groups, two in 2023 with therapist A (from Germany) –one group with 7(5) and one with 11(10) participants–, and one group in 2024 with therapist B (from the USA) with 12(11) participants (numbers in parentheses are factually attending participants).

On the first date, participants were invited 1.5 h earlier than the start of the session to conduct the interviews ‘Interview’ means here that the questionnaires were completed in an oral conversation format, with a student helper interviewing one or two of the participating women at each timepoint (when the participant preferred questionnaires were also completed self-administered, in about half of the cases). Interviews were conducted with a booklet containing (a) the demographic data, and (b) all the questionnaire-scales mentioned in the *Materials and Instruments* subsection. Participants were interviewed by helpers, mostly students of SRH International Master’s programs in dance and music therapy, which allowed us to do the interviews in their mother language or a joint language participants spoke well, and to account for persons unable to read or write. When the women arrived on the first day, we started with cake and coffee and after 40 min the therapist did a short welcome in movement with them; then, we broke off into the interviews. Each interview was conducted individually, the dyads either picked a corner of the big studio to sit down together or went back out into the quiet hallway with its sitting corners and vast special possibilities. The primary outcome measure, the RHS-15 existed in an authorized version in all languages needed, the other questionnaires had been informally translated into the interview languages by the MA-students before the start, using words to maximize understanding, and had been rehearsed to be administered in a neutral way. The duration of the interview was 20–30 min per participant.

Both therapists conducted the 90-min DMT sessions in English, with co-therapists providing periodic translations. Therapist A was German, with a very good level of English, and therapist B was from the USA, and spoke English and German. Therapists emphasized at the beginning of the sessions that this was a safe space with no harmful behaviors toward oneself or others tolerated. That each participant was in the group to regain health and that the group was called to support this goal. Therapists and study leaders agreed that DMT methods should be geared to stabilization and resource-orientation. Participants were allowed to remove their shoes for comfort and to rest as needed, just letting the therapist know. We kept the tradition of starting with coffee and cake for all sessions. Childcare was offered by voluntary DMT/MT students of SRH in the slightly separated sitting corner in the same big studio space. Before and after each session, the three-item process questionnaires were administered by the co-therapists.

Participants were interviewed with the three main scales at baseline, before and after the intervention, and at follow-up. The volunteer interviewers were international students of the Master programs in DMT and Music Therapy of SRH University Heidelberg from different countries including Australia, Iran, Singapore, Ukraine, Turkey, Taiwan and the USA. We took measures that the same interviewer was present for them throughout the trial, which worked in about 85% of the cases. For the last of the eight sessions, participants were asked to stay one hour longer (after the DMT session) to conduct the final interview. For follow-up, women of all three actual therapy groups were invited to come back to the studio in July 2024 to have a last session with therapist (from the group in 2024) and have the final interview once again (five women of group 2 and group 3 respectively participated). Every now and then an interview had to be conducted outside of the group context and was then conducted either by inviting the participant into the space at another point in time (in about 5% of the cases), or on the phone (in about another 5% of the cases). On site, about 40% of the interviews were done by the participants in a self-administered way, with the interviewers just being there for questions, 60% of the interviews were done as oral interviews in the baseline and pretest (t1), by the posttest (t3) and follow up this percentage switched to about 40% interviews and 60% self-administered questionnaires. A short focus group interview of 25 min was conducted with the therapy group of 2024 in the final session after the individual interviews [62].

The intervention: For the intervention, we worked with two certified DMT therapists (both female and Caucasian, ages 31 and 43), academically trained on MA-level within the standards of the German professional association BTD e.V. at SRH University Heidelberg and working in the region. They were funded through the research fund of the Faculty of Therapy Sciences at SRH University Heidelberg. There was no prespecified intervention protocol. Both therapists took a resource oriented-creative approach, where they checked into the needs, (cultural) possibilities, and strength of the woman in order to gear their interventions to their needs. Both therapists followed important principles of trauma-based work such as keeping the participants in the window of tolerance, working in a strength-based stabilizing manner, using grounding and mirroring techniques to counteract dissociation, and emphasizing post-traumatic growth. Each therapist worked with a main co-therapist (DMT-student that spoke the main language of the group; if group composition required it, there were up to two further DMT student interns for translations). We did not employ a pre-specified intervention protocol for three main reasons: (a) the therapists preferred to adapt the interventions to the needs of the women as they went into the sessions with completely unknown vulnerable clients, (b) there was no funding to construct an intervention protocol and provide therapist training or do preliminary interviews with the participants to get to know them and their needs beforehand, and (c) most of the eleven studies identified in the literature review, did not specify the intervention in a way that was replicable, with the exception of Harris (2007 a, b). However, Harris worked with former boy soldiers, which had different needs. For replicability, therapist A left us with a full description of her interventions (for employment in future studies), and the co-therapist of groups 1 and 3 provided us with some session impressions in English (see supplement, Appendix A). Next to methods of up- and down-regulation (Dieterich-Hartwell, 2017), relaxation, interpersonal approaches, attention-based body psychotherapy interventions (Caldwell, 2018), the Baum-circle (Koch, 2020; see supplement; Appendix B) was successfully offered as an intervention method.

### Materials and instruments

#### Setting

Regarding the setting, we were fortunate to have INTER-ACTIONS dance company as a collaboration partner, who provided us with a downtown dance studio (just across the main train station) without any costs to support our project. The studio was on the first floor, 360m^2^ in size (18 × 20 m), with a professional dance floor, windows with heavy black theatre curtains on three sides, and a small kitchen and cozy leisure corner, with a big table and sofas. Each session students, therapists, or the women themselves brought some coffee and cake to start with a get together before the start of the DMT intervention. Childcare was arranged through two students of SRH University for each of the sessions and was used in the second and third group with mostly one or two children present. Childcare was either in the same room (when the age of the children required it – there was one baby present; or when the children were drawing) or in the same building which offered vast possibilities with sitting corners in the floors and possibilities to play there. After the sessions there was a one-hour transition time until the next group came in. The dance studio space offered a great and protected atmosphere for the therapy groups altogether.

#### Materials

Regarding the materials, we had a selection of props always present in the room: chiffon scarfs in all colors, balls, hedgehog balls, bean bags, a big stretch band for about twelve persons (velvet trust band handmade by D. Avula, DMT prop shop), a card set with diverse photos of landscapes, objects or interpersonal situations, and crayons, pencils, pens and blank paper for drafts, writing, journaling and drawing as part of the sessions or thereafter; drawing paper with animals and mandalas to color for the children in childcare, and two table games for them.

#### Instruments

The psychometric instruments used in our study were: (a) the Refugee Health Screener (RHS-15) as the *primary outcome* measure [61], (b) the Posttraumatic Stress Disorder Checklist Scale (PCL-S) [60], and (c) the Depression Anxiety and Stress Scale (DASS21) [59] for measurement of adjacent symptoms.

1. In the interview *demographic data* was covered first including age, gender, nationality, professional group, residency status, marital status, children, education, living conditions, financial resources, affordable meals per day, etc.
2. The *Refugee Health Screener-15 (RHS-15)* [61], our primary outcome measure, is a screening tool for common MH symptoms for refugees 14 + years, available in 15 languages screening for depression, anxiety, trauma and stress symptoms. It uses a 5-point Likert scale from 0 ("Not at all") to 4 ("Very much"), covering three subscales: depression (4 items), anxiety (4 items), trauma (4 items) with an additional coping item (item 14), a pain item (item 1), and the stress thermometer (item 15; on a scale from 0 = no distress to 10 = extreme distress, which is treated separately, i.e. it is not entering in the sum or means of the scale summary values). Sample items include "Feeling down, sad or blue most of the time" (depression) and "Nervousness or shakiness inside" (anxiety) or "Felt emotionally numb (for example, feel sad, but can’t cry; unable to have loving feelings" (trauma). The RHS-15 has demonstrated high reliability, with *Cronbach's alpha*-values of 0.81 to 0.95 for the total score in different ethnic groups. In our study, the RHS-15 had an internal consistency of *Cronbach’s alpha* = 0.93 (computed with the values of t3).
3. The *Posttraumatic Stress Disorder Checklist Scale (PCL-S)* [60] is a 17-item self-report questionnaire designed to measure PTSD symptoms based on DSM-IV criteria, using a 5-point Likert scale from 1 ("Not at all") to 5 ("Extremely"). It covers three subscales: Reexperiencing, Avoidance, and Hyperarousal. Sample items include ‘Do you suffer from:’ "Repeated, disturbing memories, thoughts, or images of the stressful experience?", or "Feeling very upset when something reminded you of the stressful experience?" The PCL-S has demonstrated high reliability, with *Cronbach's alpha*-values of 0.86 for the total score, 0.67 for Reexperiencing, 0.69 for Avoidance, and 0.69 for Hyperarousal, and a test–retest reliability of *Pearson correlations* = 0.80 for the total score. In our study, the PCL-S had an internal consistency of *Cronbach’s alpha* = 0.88.
4. The *Depression, Anxiety, and Stress Scale-21 (DASS21)* [59] is a 21-item self-report instrument measuring depression, anxiety, and stress on three subscales containing seven items each that are rated on a four-point Likert scale ranging from 0 (never) to 3 (almost always). Sample items include "I found it hard to wind down" (stress), "I was aware of dryness of my mouth" (anxiety), and "I couldn't seem to experience any positive feeling at all" (depression). The authors report an internal consistency of *Cronbach’s alpha* = 0.90 for depression, 0.82 for anxiety, and 0.87 for stress, indicating very good reliability. Validity of the scales has been supported through confirmatory factor analysis, demonstrating adequate convergent, discriminant, and nomological validity. In our study, the DASS21 had a *Cronbach’s alpha* of 0.93.
5. The *session questionnaire*, provided at the beginning and at the end of each session by the co-therapist, contained 3-times: (a) the *stress thermometer* (0 no distress to 10 extreme distress), (b) the *coping question* from the RHS-15 (0 = high coping to 4 = low coping), and (c) a question on the *experience of beauty (*= *EoB; beauty in a holistic aesthetic sense; 0* = *no EoB to 10* = *high EoB),* and served to generate process data*.*
6. The *focus group interview*, conducted in the last session of group 3 (study group of 2024), used open questions [63]; for the analysis we employed text transcriptions in word (see supplement)^2^ and a content analysis oriented at the outcome domains of the Dunphy Outcome Framework (DOV; 68) with the predefined categories of cognitive, emotional, social, cultural, and integrative changes experienced by the women.

### Data analysis

For the statistical analysis, the alpha-level was set to *p* < 0.05. We computed a *General Linear Model (F-test)* with repeated measures for within-group differences in SPSS 20.0. A power calculation with g*power [65] for a within-group design with three repeated measures, a *p-*level of 0.05, *power* of 0.80, and expecting a medium effect size *f* = 0.30, indicated that we needed 20 participants for the critical *F*-value of 3.24. Because a pretest for sphericity resulted significant, we employed the corrected *F*-value of the Greenhouse–Geisser statistics. Pairwise comparisons (Bonferroni) were used to determine where exactly (between which two measurement points in time) the significant difference had occurred. F-tests were computed for all three main scales and their subscales (t1 to t3). *RHS-15 computation values* included Items 1–14, as suggested by the authors [61], with the stress thermometer (item 15) being treated in a separate analysis. *Follow-up* (t4) inferential statistics were computed with an additional repeated measures *F*-Test for *N* = 10, and additionally with a split data set for *N* = 5, using the pretest (t2) as the comparison group. Because of the small sample size, the follow-up analysis was done separately as an exploratory analysis. The changes per session and across sessions (process questionnaire) were computed with t-tests for related samples. An exploratory analysis of control variables was done to see influences from demographic values. A factor analysis and reliability analysis on scale homogeneity was conducted to explore how well the scales worked in our sample. All tests were computed with SPSS 20.0.

#### Missing value estimation

We estimated single missing values (six cases) by inserting the means of all remaining items on the same subscale. In addressing two missing values (one for t1 and one for t2 from different participants) from the RHS Stress Thermometer data (one item only on the RHS-15), we estimated the missing values using the equivalent of the sum value from the DASS21 stress subscale. Because of the small data set, it was not possible to estimate the missing baseline values of the four women of the first group with multiple imputation [66]. Even though *N* = 20 women would have been enough to run the analysis with full power, we preferred to keep the first group in the data set and estimated their missing values from the pretest values. The inclusion or exclusion of the four women of the first group from the data set renders no effective changes of effects.

## Results

### Sample characteristics/demographic data

Table 2 displays demographic and contextual data of our sample.

**Table 2:** Demographics of the sample

	**N**	**Percent**
Meals per day		
- 2 or less	8	33,3%
- 3 or more	16	66,7%
Decent housing	15	62,5%
Employed	15	62,5%
Nationality (*N* = 24)		
Country of residency	7	29,2%
Europe	8	33,3%
North Africa	0	0,0%
Sub-Saharan Africa	2	8,3%
Middle East	6	25,0%
Other	1	4,2%
Residential status (*N* = 24)		
Secure Residence (Permit, Asylum)	17	70,8%
Insecure Residency (Asylum Seeker, other)	7	29,2%
Education (*N* = 24)		
Never attended school	1	4,2%
Middle school	1	4,2%
Highschool	4	16,7%
Short term high education	3	12,5%
Long term high education	15	62,5%
Professional category (*N* = 24)		
Farmer, craftsman, shop	1	4,2%
Intellectual profession	2	8,3%
Employee	5	20,8%
Worker	1	4,2%
Professional training	4	16,7%
Inactive	9	37,5%
Other	2	8,3%
Resources per month (*N* = 24)		
None	2	8,3%
Under 520€	15	62,5%
520€ to 1250€	3	12,5%
1500€ to 1700€	2	8,3%
1700€ to 2600€	2	8,3%
More than 2600€	0	0,0%
Life place (*N* = 24)		
Emergency shelter	2	8,3%
Apartment	15	62,5%
Hotel	0	0,0%
Shelter	4	16,7%
Other	3	12,5%
Marital status (*N* = 24)		
Single	11	45,8%
Married	6	25,0%
Couple not living together but in the same country	2	8,3%
Divorced	5	20,8%

The majority of the women in our sample were relatively well-off, educated, with secure residency status, settled in apartments, still the financial resources were low, with some women unable to afford three meals a day; The distribution of nationalities into the three factual therapy group was as follows: *Group 1*: 3(4) Afghani women, 1 Ukrainian woman; *Group 2*: 8 Ukrainian women, 1 woman from Cameroon, 1 woman from Turkey; *Group 3*: 2 Kurdish women from Turkey, 1 Nigerian woman, 1 woman from Cameroon, 1 woman from Afghanistan, and 6 Ukrainian women. The subsample for the follow-up (*N* = 10) consisted of 6(7) Ukrainian women, 1 Nigerian woman, 1 woman from Cameroon, 1 woman from Afghanistan and 1 woman from Turkey

### Descriptive statistics

#### Main analysis

Table 3 depicts the descriptive results of the study providing means, standard deviations and N.

**Table 3:** Descriptive results of main findings Means (SD), N

	**Baseline t1 (*N* = 24)**	**Pretest t2 (*N* = 24)**	**Posttest t3 (*N* = 24)**	***Cutoff Values***^***a***^
RHS-15 Depression, Anxiety, Trauma	31,79 (11,39)	32,17 (12,41)	23,58 (13,44)**	*15/14*
PCL-S Trauma	52,46 (14,46)	50,08 (14,99)	43,67 (14,53)**	*44*
DASS21 Depression, Anxiety, Stress	63,25 (24,69)	65,17 (27,16)	53,08 (27,45)**	*45 (62 < severe)*

*N* number of participants, *SD* Standard Deviation, *RHS-15* Refugee Health Screener(15 items), *PCL-S* Posttraumatic stress disorder checklist scale (17 items), *DASS21* Depression Anxiety and Stress Scale (21 items); Maximum Sum Values are: MaxSum_RHS_ = 56; MaxSum_PCL-S_ = 85; MaxSum_DASS_ = 126; ^a^Cutoff-values: RHS-15: The manual states a clinical cutoff of 15 (Hollifield et al., 2013), which can be adjusted to 14 (Kaltenbach et al., 2018); PCL-S: The cutoff score of 44 on the French version PCL-S distinguishes between the PTSD group and control group with a high diagnostic efficacy (0.94″; Ventrureyra et al., 2002); DASS21: The cutoff value of 62, distinguishes severe from moderate values (Nazemi, 2024), the clinical cutoff is 45 (Lovibond & Lovibond, 1995)

**p<.001

**Fig. 1:**
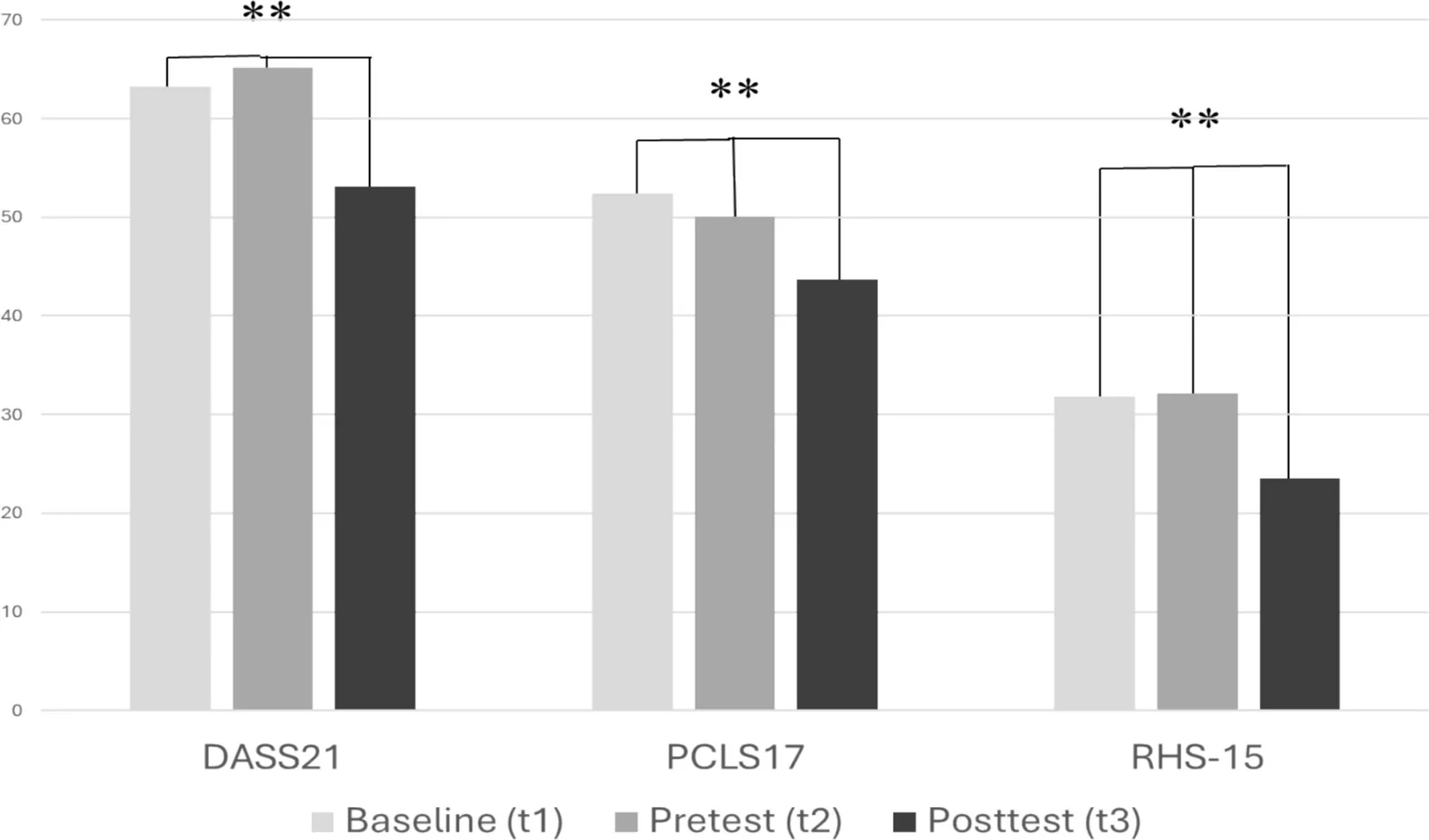
Symptom decrease on the three measures of the study. Note. **p <.01 (t3 vs t2*, and* t3 vs t1); the pairwise comparisons revealed significant differences between t1 and t3, as well as between t2 and t3

Symptoms of trauma, depression, anxiety and stress decreased on all dependent measures (RHS-15; PCL-S; DASS21). Initial values at t1 displayed severe trauma scores for 22 out of 24 women; all 24 displaying clinically relevant PTSD values; values remained high after the 8-week waiting period and decreased markedly after the 8-week intervention period, falling under the clinical cutoff score of 44 for trauma (PCL-S) [60], and under the DASS21 score of 62, differentiating severe scores of general affective distress from moderate scores. They did not fall under the clinical cutoff scores of 45 for the DASS21 [67], or of 15/14 for the RHS-15 [61, 68]. Figure 1 depicts the main findings graphically.

#### Follow-up analysis

The descriptives of the exploratory follow-up analysis (*N* = 10; Table 4) were as follows.

**Table 4:** Descriptives of exploratory follow-up analysis (comparison t2 and t4), Means (SD), N

	**Pretest t2 (*N* = 10)**	**Follow-Up t4 (*N* = 10)**	**Pretest t2 (*N* = 5**_**8-weekFU**_**)**	**8-week FU (*N* = 5**_**8-weekFU**_**)**	**Pretest t2 (*N* = 5**_**1-yearFU**_**)**	**1-year FU (*N* = 5**_**1-yearFU**_**)**
RHS-15	34,20 (12,26)	26,30 (12,25)	38,6 (10,21)	26,60 (6,46)	29,6 (13,32)	26,00 (17,21)
PCL-S	57,90 (17,16)	48,10 (15,08)	64,6 (13,28)	50,80 (9,76)	45,4 (18,85)	45,40 (19,95)
DASS21	68,80 (28,13)	51,40 (29,51)	77,6 (22,29)	59,60 (29,51)	60,00 (33,02)	43,20 (30,35)

*N* number of participants, *SD* Standard deviation, *RHS-15* Refugee Health Screener(15 items), *PCL-S* Posttraumatic stress disorder checklist scale (17 items), *DASS21* Depression Anxiety and Stress Scale (21 items); The difference of t2 and t4 was computed with a t-test for related samples and yields a difference on the 10%-level for *N* = 10; when split into the 8-week follow-up and the 1-year follow up, the 8-week follow-up shows a significant change, whereas the 1-year follow-up does not; note that the five participants for the two follow-up time points are not the same but different participants (*N* = 10 altogether); note that the absolute numbers of t4 are lower for the 1-year follow-up, but comparing the values of the five participants each to their t2 values, the difference is only significant for the 8-week follow-up (dating back to those participants among other things being more severely burdened)

### Inferential results

#### Main analysis

Table 5 depicts the inferential statistics providing Greenhouse–Geisser corrected F-values, p-values and effect sizes for total scales and subscales.

**Table 5:** Results of inferential group comparisons tests (F, p, eta2)

Scale	Subdimension	df	F (GG)	p	eta^2^
RHS-15	Total (Items 1–14)	1	9.65	.002*	.30
	Depression	1.51	9.49	.001*	.29
	Anxiety	1.39	5.35	.018*	.19
	Trauma	1.40	5.71	.014*	.20
	Stress^1^	1.65	7.35	.004*	.26
PCL-S	Total	1	10.56	.001*	.32
	Hyperarousal	1.32	6.95	.008*	.23
	Avoidance	1.74	10.24	.000*	.31
	Reexperiencing	1.86	2.97	.066	.11
DASS21	Total	1	7.45	.006*	.25
	Depression	1.46	7.88	.004*	.26
	Anxiety	1.41	2.50	.113	.10
	Stress^a^	1.55	4.41	.027*	.16

*df* degrees of freedom, *F(GG)* F-Value with Greenhouse–Geisser correction, *p* probability (significance level at *p* <.05), *eta*^*2*^ = partial eta^2^ (effect size), *RHS-15* = Refugee Health Screener(15 items), *PCL-S* Posttraumatic stress disorder checklist scale (17 items), *DASS21* Depression Anxiety and Stress Scale (21 items)

^a^Stress Thermometer (= Item 15 of Refugee Health Screener RHS-15: scale from 0–10; 0 = no distress; 10 = extreme distress; a one–item scale)

##### Pairwise comparisons

Pairwise comparisons suggest that the RHS-15 showed a mean difference of −0.606 (*p* = 0.015*) from t1 to t3, and a mean difference of −0.631 (*p* = 0.006*) from t2 to t3; the PCL-S showed a mean difference of −8.792 (p = 0.007*) from t1 to t3, and from t2 to t3 a mean difference of −9.417 (*p* = 0.005*); the DASS21 showed a mean difference of −13.167 (*p* = 0.036*) from t1 to t3, and a mean difference of −15.083 (*p* = 0.019*) from t2 to t3; and (pairwise comparison for subgroups in the supplement; Appendix C).

##### Primary outcome

RHS-15: The total *RHS-15* score decreased significantly from before to after the intervention, with *F*(1,23) = 9.65, *p* = 0.002, *eta*^*2*^ = 0.30. On the subscale-level, *depression* levels differed with an effect of *F*(1.51,23) = 9.49, *p* = 0.001, *eta*^*2*^ = 0.29. *Anxiety* levels, as measured with the RHS-15, showed significant improvement with *F*(1.39,23) = 5.35, *p* = 0.018, *eta*^*2*^ = 0.19. *Trauma symptoms* decreased significantly with *F*(1.4,23) = 5.71, *p* = 0.014, *eta*^*2*^ = 0.20, and the *stress* thermometer, showed a significant reduction of distress with *F*(1.65,23) = 7.35, *p* = 0.004, *eta*^*2*^ = 0.26. All effects favoring the intervention period over the control period.

##### Secondary outcomes

(a) PCL-S: The total score on the *PCL-S* decreased significantly from before to after the intervention, with *F*(1,23) = 10.56, *p* = 0.001, *eta*^*2*^ = 0.32, indicating overall improvement. The *hyperarousal* subdimension showed significant decrease with *F*(1.32,23) = 6.95, *p* = 0.008, *eta*^*2*^ = 0.23). *Avoidance* significantly decreased with *F*(1.74,23) = 10.244, *p* = 0.000, *eta*^*2*^ = 0.31. The reduction in *re-experiencing* exhibited a non-significant trend *F*(1.86,23) = 2.97, *p* = 0.066, *eta*^*2*^ = 0.11); (b) DASS21: The total *DASS21*-value decreased significantly after the intervention with *F*(1.55,23) = 7.45, *p* = 0.006, *eta*^*2*^ = 0.25. *Depression* levels on the DASS21 decreased markedly from t2 to t3 of *F*(1.46,23) = 7.88, *p* = 0.004, *eta*^*2*^ = 0.26. *Anxiety* showed a clear but non-significant decrease from t2 to t3 of *F*(1.41,23) = 2.50, *p* = 0.113, *eta*^*2*^ = 0.10. *Stress* levels showed significant reductions as measured by the DASS21 stress subscale, with notable decreases from t2 to t3 of *F*(1.55,23) = 4.41*, p* = 0.027, *eta*^*2*^ = 0.16. All effects favoring the intervention period.

##### Control variables

An *exploratory analysis of control variables* suggested two variables of influence: (a) Ukrainian participants benefited more than participants from other countries (*p* = 0.051), and (b) participants with a higher symptom burden on the RHS-15 benefited more than those with a lower symptom burden (*p* = 0.030) computed from a median split of symptom burden at the sum value of 32). None of the other control variables had a significant influence (all exploratory analyses see supplement, Appendix D).

##### Follow-up analysis

Follow-up (FU) analysis was exploratory, because of the small sample size. Explorations with an F-test (GLM for repeated measures), comparing t2 (pretest) and t4 (FU) values (basis of S2; RHS-15 items 1–14) yielded the results in Table 4, with *F*_t2t4_(1,8;9) = 3.36; *p*_t2t4_ = 0.064, *eta*^*2*^ = 0.27. Even if the 1-year follow-up resulted in lower symptom values, these five participants had lower values at t2 and thus the comparison with t2 was not significant, *F*_t2t4_(1;9) = 0.17; *p*_t2t4_ = 0.699, *eta*^*2*^ = 0.04, whereas the five persons at the 8-week FU had higher values at t2, which resulted in a significant decrease to the follow-up values (t4), with *F*_t2t4_(1;9) = 26.18; *p*_t2t4_ = 0.007, *eta*^*2*^ = 0.87.

##### Factor analysis and reliability analysis

Factor analyses (FA) and reliability analyses (RA) of scale homogeneity indicated very good reliabilities and explanation of variance for all outcome scales for our group of participating women.

##### Factor analysis

FA yielded an explanation of variance of 75,22% for the RHS-15, of 64,97% for the PCL-S, and of 66,40% for the DASS21. Resulting factors displayed no clear dimensionality, but a strong general factor for all three outcome measures.

##### Reliability analysis

RA yielded excellent results for all three outcome measures. Cronbach’s Alpha values of scale homogeneity resulted in 0.93 for the RHS-15, 0.91 for the PCL-S and 0.93 for the DASS21, without any item scoring less than 0.90 (FA and RA were computed with the post-test values (t3); details see supplement, Appendix E).

#### Process questionnaire

**Fig. 2:**
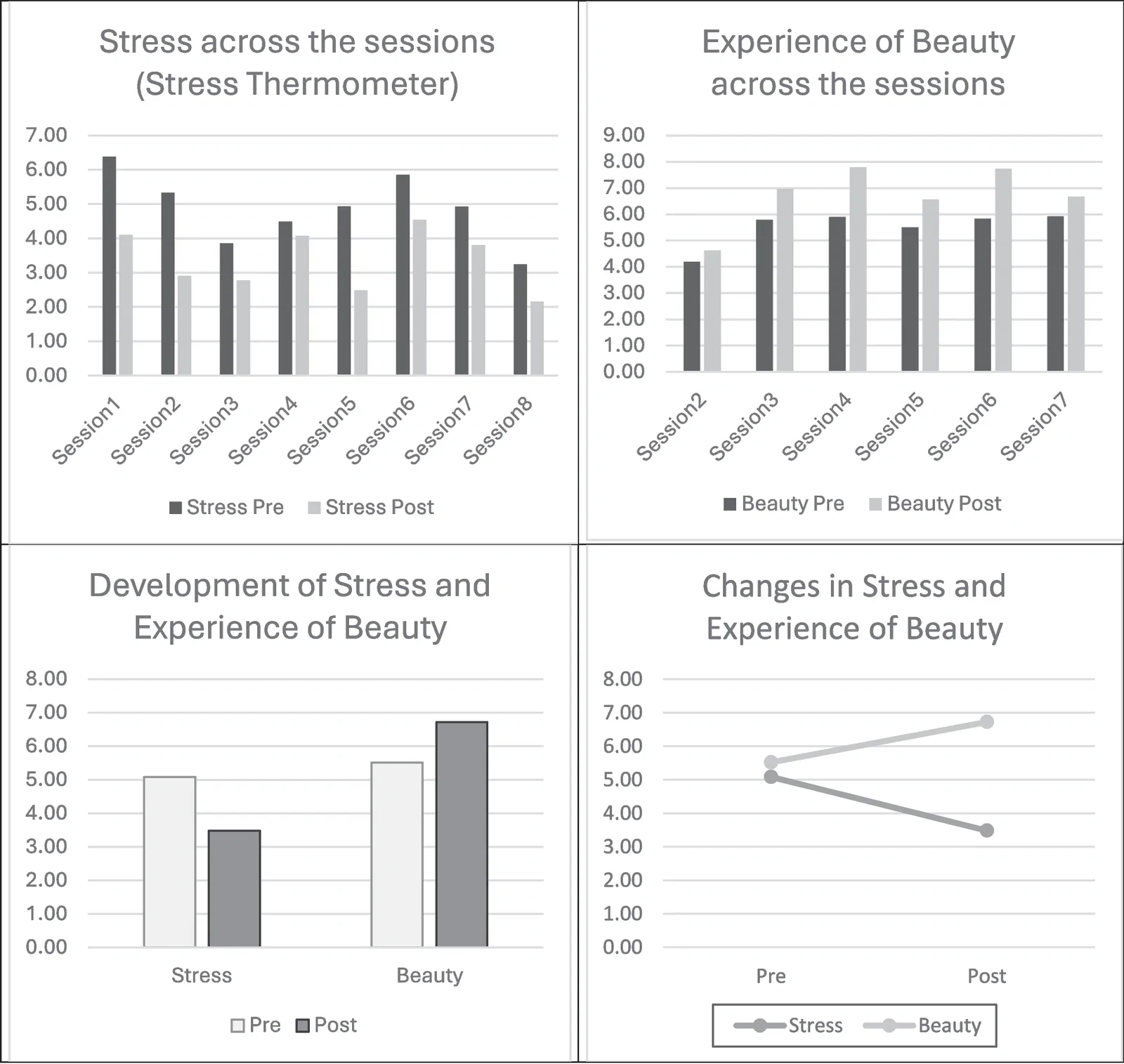
Development of stress and experience of beauty across the sessions Note. The stress thermometer was employed in the interview and in the process questionnaires (scale from 0 = no distress to 10 = extreme distress); experience of beauty was only measured in the session questionnaires, therefore, there was no pre-value in the first session; we added a post-value for the last session to the interview (scale from 1 = not at all to 10 = very high). Figures on top show the development across the sessions, bottom figures depict the development from the first/second to the last session

Before and after each session stress, coping and the experience of beauty were measured on Likert-scales. The coping scale (from ‘0’ lowest coping to ‘4’ highest coping) was used inconsistently by the participants, often inverted and inconsistent with other answers. We therefore did not include it into the analysis of pre-post data. Figure 2 shows the graphics from the process questionnaire (session means of all participants per session) for stress (0 = no distress; 10 = extreme distress) and experience of beauty (0 = no EoB to 10 = highest EoB) from all three factual therapy groups. Stress and experience of beauty after the sessions were often sig. negatively correlated in four out of seven cases (with *r* between −0.522 and −0.669, *p* between 0.038 and 0.005, and *N* between 16 and 19).

### Qualitative results

After the final session of group three and the final interview, co-therapist and second author ZN conducted a brief focus group interview with the *N* = 12 participants of therapy group 3 [62]. She was a person of confidence for the women. The transcript of the focus group interview is in the supplement (Appendix F), and excerpts in (Table 6). The purpose of the interview was to explore the changes the women experienced in their lives and that they attributed to the participation in DMT. This conversation allowed participants to express their thoughts and feelings about the DMT sessions, aiming to ensure a comprehensive understanding of the therapy's impact.

**Table 6:** Exemplary quotes of the interview (Group 3, June 2024)

Selected interview quotes of the participating women:
*“I see a lot of changes in me. Firstly, the confidence. Yeah, most times, most times when I walk on the road, I cry. Yeah. And I felt, how will I continue to move again? Like, will I be able to walk for myself, be independent, focus? And now I can stand up and look at the mirror. Before, when I look at the mirror, I just cried. I see myself in another image. Yeah. But now, I feel better, a lot of different. I can read. I can take my time to take a book and read without crying or watching a movie and without thinking about my past or just walking maybe on the streets and something just crossed my mind. I won't be able to concentrate more and yeah. Thank you. Thank you to everyone, to the We, participants, I say thank you to the leaders.”* (woman from Nigeria, 26 years, who traveled each week 2.5 h one way by train to be part of the sessions; she did not miss one of them)
*“Today I came, I was feeling pains again. Immediately I danced the pain disappeared. I had pain here, it disappeared. When Thursday is coming like this, I am very happy. The day I don't come, I am not happy. And as it is finishing, I am not happy. If it has to be continuing, continuing, I will be part of it. I would want to be because when I come here, I am fine. When I go back home, I concentrate. I thank whoever gave my name to her to put me inside the group (social worker from psychosocial center NB). I am very, very grateful that people are seeing into our worries, into our problems, making us at least mental health is the best thing so far. So I am very, very grateful and I pray that you continue with the work. To see into it that we are fine. At least that's all I have to say. Thank you very much. I'm very, very grateful. It has really changed me.”* (woman from Cameroon, 59 years)
*“As you know, I came here with suicidal thoughts and I was really, it was terrible time. I was on the medication and stuff. And when the therapy started, actually I stopped taking medication and I was afraid of this effect, when you stop it. And it really helped me to go through this period, even without tablets. (humorous) And I can't say that something drastic has changed. However, I don't want to kill myself anymore.”* (woman from Ukraine, 36 years)
*“…And even though I had a couple emotional breakdowns during the sessions, but it was really deep. And I think it was also good, because as someone mentioned, I felt safe here to actually show these emotions and live through them whatever was happening around. So thank you very much for everybody in the group for this feeling of safety. (moved) It was really cool, so thank you for that. Thank you. And I also have this crazy idea of considering dance therapy as my another education, because I've always been into psychology, into therapy. Yeah, why crazy then? Here in Germany, everything seems to be crazy when you are a refugee, I mean.”* (woman from Ukraine, 32 years)

In sum, the qualitative analysis of the interviews (following the domains of the Dunphy Outcome Framework: physical, cognitive, emotional, social, integrative and cultural [69]) indicated that the dance therapy sessions yielded a high acceptance and a profound impact on participants across various dimensions. Emotionally, participants experienced improved stability, confidence, and the ability to manage intense emotions. Cognitively, they reported increased mental clarity and insight. Socially, they built more positive relationships and enjoyed the supportive group dynamics. The experience of beauty was enhanced through self-care and aesthetic appreciation. Spiritually, participants found inner peace and emotional healing. Culturally, they adapted and integrated into new social contexts while engaging in culturally enriching activities.

## Discussion

The results of the analysis indicate significant reductions of symptoms after the intervention on the three main outcome measures: (a) the RHS-15 for stress, depression, anxiety and trauma, (b) the PCL-S for trauma, and (c) the DASS21 for stress, depression and anxiety. In sum, the data indicate substantial improvements across multiple dimensions, suggesting that the intervention was effective in mitigating symptoms of depression, stress, anxiety, and trauma among participants. Moreover, trauma symptoms of hyperarousal and avoidance were significantly reduced. The study was fully powered for medium effect sizes, which resulted for all three measurements. Even though we used a withing-group design, the changes can likely be attributed to the intervention, since participants had no other psychotherapeutic treatment during the time of the study.

### Test of the hypothesis

#### Main results

Findings of the main analysis indicate that Hypothesis H0 (there is no symptom change after DMT) needs to be rejected, and H1 (there is symptom change after DMT) is supported. Symptoms of trauma, stress, depression and anxiety were all significantly reduced after the intervention on the RHS-15 and on the PCL-S. PCL-S values fell under the clinical cutoff of 44 for trauma [60]. DASS values of depression and stress were significantly decreased, with the score falling under the mark of 62 for severe impairment [62] to moderate impairment. The RHS-15 worked well as an outcome measure in our study, with the subdimensions replicating well in a factor analysis with a variance explanation of 0.74, and with an internal consistency of *Cronbach’s alpha* = 0.93. It was beneficial to have the validated translation in each of the languages needed. However, the coping item (item 14) of the RHS-15 clearly did not work for our participants, it was often answered inconsistent with the other items, likely dating back to confusion about the poles. After completing three major symptom scales with deficit-oriented questions, the coping item (0 = able to handle (cope with) anything to 4 = unable to cope with anything) was often completed choosing the high pole for the positive one, while it was meant to be the negative (unable to cope with anything). We nevertheless kept the item in the analysis for all analyses, since the manual requires it to be added to the sum value of the scale. Without the item the effects came out clearer, thus our reported effects are a bit lower than the actual effects without item 14 would have been. We dropped the coping item in the analysis of the process questionnaire and only computed the changes in stress and experience of beauty.

#### Process questionnaire

The process data indicate reduced stress levels after each session and throughout the entire series of sessions. Additionally, there was a notable increase in the perception of beauty among participants compared to their self-assessments prior to the sessions. The coping item was dropped for the reasons stated in the paragraph above. Participants reported consistently less *stress* after the sessions than before the sessions. The focus on the holistic appreciation of the *experience of beauty* may have had an important impact on our participants. Improvements in self-care, aesthetics, and overall self-image were reported in the final interview, i.e. participants made significant changes at home, such as cleaning, decorating, adding flowers, and enhancing their living environments. According to Koch et al. (2016), *aesthetic experience* is considered an important therapeutic factor for effects of arts therapies on health. By asking about the experience of beauty, the intervention may have empowered participants to discover and appreciate their inner and outer beauty, possibly promoting a sense of self-worth and well-being.

#### Interview data

The focus group interview after the last session suggest that DMT can be an effective intervention for refugee groups of diverse cultural and language backgrounds suited to create *holistic well-being*, addressing emotional, cognitive, social, aesthetic, spiritual, and cultural needs in a supportive group setting. In the last session, we handed out addresses for further support: professional psychotherapy, cultural offers in the dance studio and beyond, and self-help groups. The last two sessions left us with a clear feeling that participants would have appreciated and benefitted from receiving more session of DMT. While there was a wish to continue, the funding for the study therapists had ceased.

### Clinical implications

We announced the study as “Dance Therapy for traumatized refugee women” to our collaboration partners. The social workers of the city administration, NGOs and PSC Nordbaden sent us ten women altogether. The rest of the women came almost exclusively via the WhatsApp-group of Ukrainian women in Heidelberg. All participating women were self-defined traumatized which was confirmed by our clinical measures. The symptoms-scores of the PCL-S reported at baseline suggested that 22 out of the 24 participating women had clinically severe PTSD symptoms (the highest category), the remaining two also had clinically relevant PTSD symptoms (the second highest category). Symptoms remained unchanged after the 8-week-waiting period, then decreased markedly after the 8-week intervention, dropping under the PCL-S clinical cutoff-score of 44. The effect is likely attributable to DMT, since neither woman received any other psychotherapy treatment. The unfortunate fact that only below 10% of the refugees in need receive mental health treatment in Germany created almost ideal conditions for our study, because due to this fact, DMT was the only intervention the women received.

The clinical significance of the study is high and may be more relevant to the practice in a medical care context than the statistical significance; according to Shama [70] “Clinically significant findings are those which improve medical care resulting in the improvement of individual's physical function, his/her mental status, and ability to engage in social life”. Whereas statistically significant results, defined by the p-value and effect size resulting from a statistical hypothesis test, need not necessarily improve the quality of life of the affected persons. The individual interviews for completion of the questionnaire and the focus group interview at the final session showed that the intervention was acceptable and feasible for all participants, reduced their MH symptoms and reportedly improved their quality of life. External validity and applicability of the trial findings are high, the trial has direct practice implications (see Table 7), with benefits clearly outweighing harms. Our study suggests that DMT is a low-threshold and highly cost-saving intervention, compared to standard psychotherapy for PTSD that can be flexibly adapted to cultural and individual needs and contexts.

Cultural adaptability is, for example, high in the Baum-circle technique, where each participant brings their own music and choses their own movement to genuinely express themselves. Based on the nonverbal character of the techniques employed, mirroring and sharing in movement and music brings the groups into cohesion and mutual support, cultural and pan-cultural/universal elements are readily integrated in an emotionally meaningful way. Sometimes –if experienced as ideosynchratic by the participant initiating the movement–, cultural or individual symbols in music or movement are explained to the group and the therapist; but many times no words are needed. The technique does not necessarily require a verbal procession part in order to be experienced as healing.

Our findings are consistent with many of the findings in the studies reviewed, for example, the ones of Verreault (2017) in her (*N* = 8) study on DMT for traumatized refugee women in the Netherland, where she concludes that “a key finding was that DMT provided a shared safe psychological space for self-expression among this vulnerable population and can be incorporated into a resilience-based treatment program with adaptations for context.” The findings of our study are generally in line with the findings and conclusions of the qualitative studies by clinical practitioners and of the reviews on these studies suggesting a high degree of converging evidence for beneficial effects of DMT for refugees.

### Limitations

Important limitations of this study consist in:

- *the small sample size*; however, while it was small, it was fully powered for medium effect sizes, which were obtained.
- *Self-selection* (self-chosen participation) and thus potential motivational bias. We utilized a convenience sample selecting participants who were readily available, with clear self-selection in most of them: 60% came completely self-referred, 40% came through the transferal or recommendation of their social workers; in all of the cases, it was the women’s voluntary decision to participate. The attraction of women drawn to -or at least not resistant to- dance (self-selection bias) could have resulted in an overestimation of the results.
- *Biased diversity* of the sample: almost 60% of the participants were Ukrainian, a variable that was correlated with the self-selection/motivational bias. Exploratory analysis of control variables suggested differences between nationalities, notably that Ukrainians benefited more from the sessions. This might be due to cultural factors, or to the fact that their legal and financial status in Germany is better than that of other refugees, or the higher cultural (feeling of) closeness to the Western therapists; maybe also due to the strong community with other Ukrainian women as the majority in two of the groups; yet the only Ukrainian woman in the first group also benefited pronouncedly. Reasons for this difference, in the end, remain speculative.
- *Design Issues.* While it was originally intended, we were unable to employ a random sampling method in a between-group design. According to Gravetter and Forzano (2003), a within-group design may have disadvantages [71], for example, it is usually difficult to determine whether changes are due to the treatments or time-related factors. However, in our study the women were taking part in direct succession in the waiting and in the intervention group and had no other mental health treatment at the side. Next to the lack of possibility of blinding the interviewers in a single-group design, effects could therefore have occurred on the basis of the natural passing of time alone, which seriously limits the interpretability of results. It is also possible that any intervention would have increased the MH of the women in this situation, just because of being seen and addressed (cf. the Hawthorne effect, [72, 73]). The design change affects the ability to attribute outcomes to the DMT intervention rather than to confounding factors such as time, attention, or expectancy effects. Thus an RCT is an important next step. However, RCTs also have their problems, for example, in traumatized populations RCTs can actually increase trauma and stress symptoms, e.g. if persons who want to come to treatment together need to be separated by randomization procedures. Barriers to conduct RCTs with traumatized persons include their high vulnerability which results in only few RCTs being conducted, and rarely by small subjects such as CATs.
- *Problems with scales*: there were limitations regarding the translations of scales. We partly worked with unauthorized translations of scales, mostly orally translated by native speaking international students from the Master programs in DMT and music therapy at SRH University, which was part of the concept. The only questionnaire that existed in all necessary languages in an officially translated and validated version, was our primary outcome measure, the RHS-15 [61]. The Ukrainian version of the DASS21 and the PCL-S had been translated by a Ukrainian translator before the start of the study, however it was not an official, validated or pretested version of the scale, it was also not translated back and forth, as required in the translation process of psychometric scales (mainly because of missing resources). We employed the Ukrainian translation in interview form by Ukrainian speakers and as in self-administrated questionnaire, making it comparable to the other interviews conducted with the official English version of the questionnaires (containing translation remarks of the interviewers), but inconsistent with the oral interviews. The same issues apply to the Farsi/Persian version of the DASS21 and the PCL-S translations, where interviewers noted that certain expressions might not have direct equivalents in Persian, or the same word might be used with different meanings. In the end, for the DASS21 and the PCL-S our best option was to choose the oral interview form to clarify language and content-related questions as the interviews unfolded. Another reason to choose the interview form was to account for the high percentage of illiterate persons among the refugee population. However, in the end, we did not have any illiterate persons in our sample. Note that adapting questionnaires into interviews introduces a high risk of bias, particularly given the lack of possibility of interviewer blinding in a single-group design, and the involvement of DMT students in conducting the interviews (expectation bias).
  Moreover, as described above, the coping item of the RHS-15 did not work well as a measure. Next to the reason with the inconsistent use of the Likert-scale, because it was the only item inversely poled, described above, some participants had difficulty understanding the concept of ‘coping’, and sometimes also of the terms “handle” and “cope”, which had been observed as challenges before [74]. In response to these challenges, the authors created a shorter version of Refugee Health Screener without the coping item [75]. We still decided to keep the coping item in the sum value, because it did not cause any significant change compared to the test or the reliability without the item in the scale, however, to take the item out of the sum value would have been also justified and would have resulted in slightly clearer results in favor of H1.
  Generally, the scales were employed mostly by therapists in training -with the exception of two of them which were conducted by a psychologist (first author SK)-, thus the criteria for a clinical diagnostic interview were not fulfilled. Because of funding reasons this was not otherwise possible. Yet, reliability values indicate that scales where used reliably by the women of the sample.
- *Expectation bias: Expectations* for desired outcome may have caused biases in the therapists, the co-therapists, interviewers, study leaders and the participants themselves [76]. Therapists may have transmitted their beliefs of the benefits of the therapy in symptom reduction to participants, and participants may have caught on to this expectation; in clinical processes, expectancies are an important therapeutic factor we want to activate to take full advantage of the treatment, whereas in a controlled study, expectancy effects need to be critically discussed. The interviewing DMT students could have transmitted their own positive expectations to the participants, particularly, because in therapy they are trained to actively work with expectations, for the research they were instructed to stay emotionally neutral, however, they may have not been completely successful. In sum, expectation bias may have led to an overestimation of the results.
- *Social desirability* answers from the side of the women is another possible bias, for example, to please those that provide you with a free treatment. On the other hand, many women were not familiar with research procedures and seemed to take one question at a time and to answer them in a spontaneous and authentic way.
- *Lack of reliable follow-up data*: Additionally, investigating the long-term effects of DMT on psychological outcomes would provide valuable insights into the sustainability of the benefits observed. We suggest that follow-ups are consistently included in further designs to assess long-term effects. In this study, we made a start to gather first exploratory follow-up data from a subsample of our participants.
- *Strength:* A notable strength of this research was the privileged circumstances in which it was conducted. These included the presence of multilingual SRH international students completing elective hours, access to a free dance studio conveniently located next to the train station, and funding for the study therapist from SRH as well as financial support for the researchers from Alanus University. A further strength is the diversity of both the international team and the participants. This diversity enriched the study and provided a broader perspective on the effectiveness of DMT across different cultural contexts. The inclusion of an international team facilitated a more inclusive and culturally sensitive approach to the intervention (it was not “the Germans” organizing something for the refugees, but an internationally diverse group of researchers with similarities of one or the other kind to the participating refugee group), while the varied backgrounds of the participants ensured that the findings were not limited to a single demographic. Future research should continue to explore these aspects.

### Future studies

Future studies are called to contribute well-designed rigorous trials, such as between group-designs and randomized designs with follow-ups adhering to the CONSORT-statement to increase the knowledge and the evidence-level of studies on DMT for refugees. To address the identified methodological barriers, standard instruments that do not exist in the participants’ mother language will need to be thoroughly translated and pretested with time and care. A between-group design should be the minimum standard of a next study building upon our study. Monitoring treatment adherence, adverse events and acceptance of treatment are recommended. These are important steps for the professional anchoring of DMT in trauma treatment, which can also advance the inclusion of DMT into the medical guideline for trauma treatment, and thereby augment the presence of DMT in the clinical treatment of affected patients.

Potential obstacles to developing robust evidence in creative arts therapies for trauma treatment result from access to and requirements of the medical system. DMT is challenged to gain funding for larger controlled trials needed to establish evidence. However, medical doctors rarely grant DMT researchers access to their PTSD patients. This is understandable in such a vulnerable patient group, on the other hand, the same medical system requires DMTs to conduct clinical RCTs with active control groups – a requirement often beyond realistic possibilities of completion.

In the funding policies, CATs proposals may be assessed by either culture-based or health system-based reviewers with different criteria and only partial understanding of the proposed integrative methods of CATs [34]. Reviewer in creative arts therapies proper are missing in the system as to the young discipline and the high workload at the universities of applied sciences, where CATs are mostly anchored in Germany, with no resources for attracting the professional research teams that would be needed to develop a grant application suited to generate reliable evidence. The studies on DMT and refugees mentioned here were mostly conducted by single investigators, often students, on a small or no budget, as was the case for our study. This creates serious barriers for the field to move forward.

Given that Ukrainian women benefited more than other nationalities (*p* = 0.051), it would be interesting to investigate reasons for this phenomenon. It raises, for example, important questions about the *cultural specificity of the interventions*. The German and US-American therapist, being culturally closer to Ukrainian refugees than any other group, could have called forth an implicit cultural bias from both the side of the therapist as well as the side of the women. Implicit, because this topic did not appear in any of the otherwise open interviews or feedback rounds. Also, the fact that the change in *anxiety* only turned out significant in the RHS-15 data and not in the DASS21, deserves mention and more investigation. Future research should also focus on investigating mechanisms of change for DMT, and other CATs. The review of de Witte et al. (2021) has shown that *embodiment, concretization and symbolic communication* are three mayor therapeutic factors of CATs. Future studies in the field of DMT for trauma could address questions such as ‘Does embodiment contribute to decrease avoidance?’, ‘Is concretization and symbolic expression effective to down-regulate hyperarousal?’, and ‘Can the aesthetic experience reduce re-experiencing?’.

A *list of practical recommendations* for further studies on the topic is shown in (Table 7).

**Table 7:** Practical recommendations for future studies

List of Recommendations
- Plan enough recruitment time. The refugee population is hard to reach, if you are not in direct contact, make use of multipliers and persons of trust such as their social workers, staff of the PSCs etc. to communicate your offer personally
- Childcare should be offered to not lose the mothers for the study
- An informal coffee and cake time can open each session. The women are able to sit and chat for a bit, or to contribute something to a joint good atmosphere from their side
- With eight sessions, the duration of the intervention felt too short for the women in their and our view. We would recommend at least ten sessions for future studies
- Next to techniques of up- and down-regulation, the Baum-circle worked well as a technique for this population, since it gave the women a chance for personal as well as cultural sharing, and self-determination on how much, what and how they wanted to share with the group in movement and music
- It worked well to give the women a choice of whether they wanted to have the interview or to self-administer the questionnaire. If the sample is big enough, subgroup analyses can be used to explore differences of the two versions
- The RHS-15 is a recommendable tool, it is short, understandable (except for item 14) and exists in most major flight languages in a validated version. Item 14, the coping item, should in the long run be replaced by a better understandable item
- Next to deficit- or symptom-oriented scales, we encourage research colleagues to increasingly apply scales of protective factors such as resilience, self-efficacy, and wellbeing
- Based on our data, incorporation of DMT into existing refugee support programs can be recommended, for example to add options of nonverbal expression suited to accomplish symptom reduction

## Conclusions

In this controlled clinical trial, in the absence of other therapies for the participants, DMT has likely caused a decrease of trauma, stress, depression, and anxiety symptoms for traumatized refugee women from diverse cultural backgrounds. Due to numerous limitations (small N, within-group design, Ukrainian over-representation, no standardized intervention protocol, non-validated scale translations for secondary measures, reliance on student translators in interview format, etc.), the evidence reported here is suggestive not conclusive and makes the results of this study preliminary. Particularly self-selection bias and expectation bias may have led to an overestimation of results. More rigorous randomized controlled trials are needed to expand upon the findings of this study, establish a more reliable causality, and thus increase the evidence level. Our study is, nevertheless, an important steppingstone on the way to more rigorous evidence-based research on DMT for refugees. Despite the limitations, it shows that DMT is a cost-effective low threshold intervention with a high cultural adaptability, potentially suited to reduce trauma and adjacent symptoms, and acceptable and feasible for participants of diverse cultural background. In times of increasing global conflicts and wars, we are expecting rather more than less forceful displacement and migration in the future. Trauma and related symptoms are likely to increase or remain. The need for therapeutic interventions that do not rely on verbal techniques only is expected to rise. DMT, relying on body movement as a universal language, has the potential to make a difference in the individual lives of traumatized women as well as on the societal level in programs supporting refugee health. It transcends linguistic and ethnic barriers as an accessible intervention for individuals from diverse backgrounds. Moreover, the group-based nature of the intervention not only addresses individual needs but also fosters strong social support and cohesion among participants. This aspect, pertaining to clinical significance, is crucial for the mental health and well-being of refugees who often experience social isolation. The universality of body movement and the experience of beauty have the potential to foster a sense of unity and wholeness, proposing the use of DMT to promote psychological wellbeing and symptom decrease across cultures and communities.

## Supplementary Information

Supplementary Material 1.

## Data Availability

Data and materials will be made available upon request by sabine.koch@alanus.edu.

## Abbreviations

ADTA: American Dance Therapy Assoziation
BAfF: Bundesweite Arbeitsgemeinschaft der Psychosozialen Zentren für Flüchtlinge und Folteropfer
BP: Body Psychotherapy
BTD: Berufsverband der TanztherapeutInnen Deutschlands, e.V.
CATs: Creative Arts Therapies
CONSORT: Consolidated Standards of Reporting Trails
DASS21: Depression Anxiety and Stress Scale (21 items)
DMT: Dance Movement Therapy
DM Therapist: Dance Movement Therapist
FU: Follow-Up
MH: Mental Health
PCL-S: Posttraumatic Stress Disorder Symptom Checklist (17 items)
PTSD: Posttraumatic Stress Disorder
RCT: Randomized Controlled Trial
RHS-15: Refugee Health Screener (15 items)
SPSS: Statistical Package für Social Sciences
UNHCR: Unites Nations High Commissioner for Refugees
WHO: World Health Organization

## Footnotes

1 We collaborated with the psychosocial center for refugees and torture victims PSC Nordbaden, and social workers of the city administration, the regional administration, the churches and NGOs for recruitment. We cooperated with an EU-student initiative (EFPSA) under the lead of C. Tomaszewski in France, using a joint assessment booklet (containing the Depression Anxiety and Stress Scale (DASS21) [59], the Posttraumatic Stress Disorder Checklist Scale (PCL-S) [60]) and demographic variables), adding a unique *primary outcome* to the German study, the Refugee Health Screener RHS-15 [61]. Ethics of the study plan were approved by the ethics board of Université de Bourgogne/Franche-Comté, France, with the ethics vote number CERUBFC-2021-11-09-035; on this basis, an ethics exempt was granted by the ethics committee of SRH University Heidelberg, Germany, on 20-12-2022. The study adheres to the CONSORT guidelines for clinical trials.

2 The focus group interview of group 3 was conducted by second author ZN [56]; focus group Interviews with groups 1 and 2 were conducted in 2023 and analyzed by Cheang [64].
