# Supplementary material for: The effect of dance movement therapy on traumatized refugee women – a clinical controlled trial
Source: BMC Complement Med Ther. 2026 Sep 25;26:277. doi: 10.1186/s12906-026-05299-1 (PMC13615640; doi:10.1186/s12906-026-05299-1)
Supplement: Supplementary file 1 [file 12906_2026_5299_MOESM1_ESM.docx]

**Appendix A**

**Intervention examples and selected session impressions of the co-therapist (ZN)**

**Embodiment and Molding of Shapes**

The session commenced with an exploration of the embodiment and molding of shapes. Participants engaged in activities designed to internalize and express various shapes through their bodies, both individually and as a cohesive group.

**Embodiment of Concepts through Watching Photos**

Building upon the theme of embodiment, participants were tasked with embodying concepts inspired by viewing photographs. This involved both individual and group activities, emphasizing the translation of ideas into physical representations.

**Dance Train with Mirroring**

A significant portion of the session was dedicated to the "Dance Train" exercise, where participants mirrored each other's movements standing in two lines in front of each other (like the railways of a train). This activity fostered a sense of unity and synchronicity, as individuals attuned to the physical expressions of their peers, creating a fluid and interconnected dance sequence.

**Baum Circle**

The Baum Circle exercise was introduced to further enhance group cohesion and individual expression. Each participant voluntarily chose a piece of music and expressed her feelings through dance while the others mirrored her movements. Following this expressive performance, the group sat together to discuss the chosen music's significance. Each participant shared why she selected that particular music, its special meaning for her, and her emotional experience while expressing herself through it. Other participants and the therapist provided feedback, sharing how they felt while mirroring and the emotions evoked through the shared dance experience.

**Embodiment of Concepts and Drawing**

Participants were then guided to embody specific concepts, translating these ideas into drawings. This artistic process was followed by embodying the drawings themselves, bridging the gap between visual art and physical expression. The transition from concept to drawing to embodiment facilitated a multi-layered exploration of creative expression.

**Zoo Dancing: Inspired by Animal Photos**

In a playful yet insightful activity termed "Zoo Dancing," participants drew inspiration from various animal photographs. They embodied the movements and characteristics of different animals, enriching their dance vocabulary and fostering a deeper connection with the natural world.

**Feminine-Masculine Energy Activation**

Participants then explored the embodiment of feminine and masculine energies by engaging with the concepts of direct versus indirect, sensing versus feeling, and fast versus slow movements. This exercise took place in a circle, allowing each participant to experience and express both types of energies. After embodying both energies, participants chose to align with either the masculine or feminine energy group, based on their current energy state.

In these groups, participants selected music and created movements that embodied their chosen energy. The session culminated in a performance where each group presented their choreography to the other. Following the performances, everyone gathered to share their individual and group experiences, discussing how embodying and expressing these energies influenced their movements and emotions.

**Goodbye Ritual**

The session concluded with a carefully crafted goodbye ritual, providing closure and reinforcing the sense of community established throughout the activities.

_________________________________________________________________

**Aggression Management Exercise**

A notable incident occurred when one participant expressed a need to let her anger and pressure out in the form of shouting. This spontaneous declaration resonated with the group, leading to a collective decision to incorporate shouting into the sessions. For two consecutive sessions, participants engaged in this cathartic exercise, using shouting as a means to release pent-up pressure and anger. This shared experience not only alleviated individual stress but also strengthened group cohesion, creating a more unified and supportive environment.

# **Appendix B**

**The Baum Circle as an example of a DMT intervention method for trauma treatment**

In the Baum-circle (Baum, 1999; Koch & Harvey, 2012), one person in free movement improvisation, to a self-selected piece of meaningful music, is followed by the entire therapy group in movement [89, 78]. Persons initiating may employ a form of Authentic Movement, just listening inside and expressing themselves (often with their eyes closed), or go into full contact and interaction with the group (or anything in between). Positive as well as negative body memories can arise. Positive body memories can be used as resources, to work on strengths and skills of the patient. Negative body memories can be further addressed in the group, or in single therapy sessions, to work -for example- on the nonverbal narrative of the issue with the goal to move on. The person initiating determines what, how much and how deep they want to go sharing aspects of their state and stories in movement. From the beginning, each member of the group with their body follows the movements and movement quality of the person initiating. The group members do not need to go into movements that are too complicated or fast for them to follow; the important thing to pick up is the quality of movement to resonate with that quality in order to get a sense of the physical state, emotional message, and ideas behind what the mover conveys, and what may evolve in the process of moving and sharing. In the end, the group sit down in a circle to process the movement material, emotions and thoughts. In a safe space, the format provides a stage for everyone, a place to share and be seen in one’s vulnerability and strengths, and be enriched with group feedback (providing mirror, understanding and corrective) and diverse perspectives toward the therapeutic goals.

Movement mirroring has similar therapeutic implications to verbal mirroring as described by Rogers in client-centered therapy [90] (Rogers, 1951). It serves the goal of establishing rapport and empathy (using emotional contagion and kinesthetic empathy); it conveys respect, acceptance, flow experiences, and reality testing opportunities to the participants. Developmental stages of mirroring [88] have recently been described by Eberhard (2012), providing a theory that helps the therapist support the client to move toward therapeutic goals. In basic research [92] Feniger-Schaal et al. (2018), using the mirror game as a technique, have demonstrated that aspects of mirroring in movement are a behavioral correlation of attachment style.

# **Appendix C**

Pairwise Comparisons for total and subdimensions of measures

| **Measure** |  |  |  |  | **Comparison** | **Mean Difference** | **Sig.** |
| --- | --- | --- | --- | --- | --- | --- | --- |
| **DASS21** | Total |  |  |  | t3 vs t1 | -13.167 | 0.036* |
|  | Total |  |  |  | t3 vs t2 | -15.083 | 0.019* |
|  | Depression |  |  |  | t1 vs t2 | -1.333 | 0.923 |
|  |  |  |  |  | t1 vs t3 | 6.250 | 0.045* |
|  |  |  |  |  | t2 vs t1 | 1.333 | 0.923 |
|  |  |  |  |  | t2 vs t3 | 7.583 | 0.009* |
|  |  |  |  |  | t3 vs t1 | -6.250 | 0.045* |
|  |  |  |  |  | t3 vs t2 | -7.583 | 0.009* |
|  | Stress |  |  |  | t1 vs t2 | 0.583 | 1.000 |
|  |  |  |  |  | t1 vs t3 | 4.417 | 0.079 |
|  |  |  |  |  | t2 vs t1 | -0.583 | 1.000 |
|  |  |  |  |  | t2 vs t3 | 3.833 | 0.125 |
|  |  |  |  |  | t3 vs t1 | -4.417 | 0.079 |
|  |  |  |  |  | t3 vs t2 | -3.833 | 0.125 |
|  | Anxiety |  |  |  | t1 vs t2 | -1.167 | 0.765 |
|  |  |  |  |  | t1 vs t3 | 2.500 | 0.601 |
|  |  |  |  |  | t2 vs t1 | 1.167 | 0.765 |
|  |  |  |  |  | t2 vs t3 | 3.667 | 0.220 |
|  |  |  |  |  | t3 vs t1 | -2.500 | 0.601 |
|  |  |  |  |  | t3 vs t2 | -3.667 | 0.220 |
| **RHS-15** | Total |  |  |  | t3 vs t1 | -0.606 | 0.015* |
|  | Total |  |  |  | t3 vs t2 | -0.631 | 0.006* |
|  | Depression |  |  |  | t1 vs t2 | 0.073 | 1.000 |
|  |  |  |  |  | t1 vs t3 | 0.802 | 0.009* |
|  |  |  |  |  | t2 vs t1 | -0.073 | 1.000 |
|  |  |  |  |  | t2 vs t3 | 0.729 | 0.009** |
|  |  |  |  |  | t3 vs t1 | -0.802 | 0.009* |
|  |  |  |  |  | t3 vs t2 | -0.729 | 0.009* |
|  | Anxiety |  |  |  | t1 vs t2 | -0.146 | 0.525 |
|  |  |  |  |  | t1 vs t3 | 0.417 | 0.160 |
|  |  |  |  |  | t2 vs t1 | 0.146 | 0.525 |
|  |  |  |  |  | t2 vs t3 | 0.563 | 0.037* |
|  |  |  |  |  | t3 vs t1 | -0.417 | 0.160 |
|  |  |  |  |  | t3 vs t2 | -0.563 | 0.037* |
|  | Trauma |  |  |  | t1 vs t2 | -0.063 | 1.000 |
|  |  |  |  |  | t1 vs t3 | 0.563 | 0.099 |
|  |  |  |  |  | t2 vs t1 | 0.063 | 1.000 |
|  |  |  |  |  | t2 vs t3 | 0.625 | 0.026* |
|  |  |  |  |  | t3 vs t1 | -0.563 | 0.099 |
|  |  |  |  |  | t3 vs t2 | -0.625 | 0.026* |
|  | Stress Thermometer |  |  |  | t1 vs t2 | 0.091 | 1.000 |
|  |  |  |  |  | t1 vs t3 | 2.295 | 0.012* |
|  |  |  |  |  | t2 vs t1 | -0.091 | 1.000 |
|  |  |  |  |  | t2 vs t3 | 2.205 | 0.032* |
|  |  |  |  |  | t3 vs t1 | -2.295 | 0.012* |
|  |  |  |  |  | t3 vs t2 | -2.205 | 0.032* |
| **PCLS17** | Total |  |  |  | t3 vs t1 | -8.792 | 0.007* |
|  | Total |  |  |  | t3 vs t2 | -9.417 | 0.005* |
|  | Reexperiencing |  |  |  | t1 vs t2 | -1.000 | 1.000 |
|  |  |  |  |  | t1 vs t3 | 2.000 | 0.318 |
|  |  |  |  |  | t2 vs t1 | 1.000 | 1.000 |
|  |  |  |  |  | t2 vs t3 | 3.000 | 0.046* |
|  |  |  |  |  | t3 vs t1 | -2.000 | 0.318 |
|  |  |  |  |  | t3 vs t2 | -3.000 | 0.046* |
|  | Avoidance |  |  |  | t1 vs t2 | -0.833 | 1.000 |
|  |  |  |  |  | t1 vs t3 | 4.417 | 0.000* |
|  |  |  |  |  | t2 vs t1 | 0.833 | 1.000 |
|  |  |  |  |  | t2 vs t3 | 5.250 | 0.003* |
|  |  |  |  |  | t3 vs t1 | -4.417 | 0.000* |
|  |  |  |  |  | t3 vs t2 | -5.250 | 0.003* |
|  | Hyperarousal |  |  |  | t1 vs t2 | -0.792 | 0.333 |
|  |  |  |  |  | t1 vs t3 | 2.292 | 0.124 |
|  |  |  |  |  | t2 vs t1 | 0.792 | 0.333 |
|  |  |  |  |  | t2 vs t3 | 3.083 | 0.009** |
|  |  |  |  |  | t3 vs t1 | -2.292 | 0.124 |
|  |  |  |  |  | t3 vs t2 | -3.083 | 0.009 |

Note: *p*=0.05; DASS21= Depression Anxiety and Stress Scale (21 items), PCLS17= Posttraumatic stress disorder checklist scale (17 items), RHS-15= Refugee Health Screener(15 items).

# **Appendix D**

**Explorative analysis of influence of control variables**

# **Appendix E**

**Results of Reliability Testing**

| Reliabilitätsanalyse: Cronbach's Alpha values for scale homogeneity | | | | |
| --- | --- | --- | --- | --- |
|  |  |  |  |  |
| **Reliabilität** | |  |  |  |
|  |  |  |  |  |
| **Skala: ALLE VARIABLEN RHS-15** | | | |  |
|  |  |  |  |  |
| **Zusammenfassung der Fallverarbeitung** | | | |  |
|  | | N | % |  |
| Fälle | Gültig | 24 | 100,0 |  |
|  | Ausgeschlossena | 0 | ,0 |  |
|  | Gesamt | 24 | 100,0 |  |
|  |  |  |  |  |
|  |  |  |  |  |
| **Reliabilitätsstatistiken** | |  |  |  |
| Cronbachs Alpha | Anzahl der Items |  |  |  |
| ,930 | 13 |  |  |  |
|  |  |  |  |  |
|  |  |  |  |  |
| **Item-Skala-Statistiken** | | | | |
|  | Skalenmittelwert, wenn Item weggelassen | Skalenvarianz, wenn Item weggelassen | Korrigierte Item-Skala-Korrelation | Cronbachs Alpha, wenn Item weggelassen |
| t3_RHS_Item 1_muscles | 20,46 | 145,389 | ,722 | ,924 |
| t3_RHS_Item 2_feeling down | 20,08 | 147,558 | ,763 | ,923 |
| t3_RHS_Item 3_thinking too much | 19,92 | 144,428 | ,660 | ,925 |
| t3_RHS_Item 4_helpless | 20,25 | 143,935 | ,818 | ,921 |
| t3_RHS_Item 5_scared | 20,63 | 145,114 | ,618 | ,927 |
| t3_RHS_Item 6_faintness | 20,88 | 144,810 | ,642 | ,926 |
| t3_RHS_Item 7_nervousness | 20,54 | 141,824 | ,750 | ,922 |
| t3_RHS_Item 8_restless | 20,75 | 147,152 | ,571 | ,928 |
| t3_RHS_Item 9_crying | 20,04 | 139,259 | ,676 | ,925 |
| t3_RHS_Item 10_reliving trauma | 20,54 | 139,042 | ,763 | ,922 |
| t3_RHS_Item 11_physical reactions | 20,46 | 137,129 | ,775 | ,921 |
| t3_RHS_Item 12_emotionally numb | 20,79 | 141,911 | ,644 | ,926 |
| t3_RHS_Item 13_jumpier | 20,17 | 143,884 | ,602 | ,928 |
|  |  |  |  |  |
|  |  |  |  |  |
| **Skala: ALLE VARIABLEN PCL-S** | | | |  |
|  |  |  |  |  |
| **Zusammenfassung der Fallverarbeitung** | | | |  |
|  | | N | % |  |
| Fälle | Gültig | 24 | 100,0 |  |
|  | Ausgeschlossena | 0 | ,0 |  |
|  | Gesamt | 24 | 100,0 |  |
|  |  |  |  |  |
|  |  |  |  |  |
| **Reliabilitätsstatistiken** | |  |  |  |
| Cronbachs Alpha | Anzahl der Items |  |  |  |
| ,913 | 17 |  |  |  |
|  |  |  |  |  |
|  |  |  |  |  |
| **Item-Skala-Statistiken** | | | | |
|  | Skalenmittelwert, wenn Item weggelassen | Skalenvarianz, wenn Item weggelassen | Korrigierte Item-Skala-Korrelation | Cronbachs Alpha, wenn Item weggelassen |
| t3_D_Q1_disturbing memories | 40,83 | 180,058 | ,792 | ,902 |
| t3_D_Q2_disturbing dreams | 41,75 | 188,283 | ,641 | ,907 |
| t3_D_Q3_reliving it | 41,25 | 184,022 | ,731 | ,904 |
| t3_D_Q4_feeling upset | 40,75 | 178,891 | ,763 | ,902 |
| t3_D_Q5_physical reactions | 41,08 | 188,775 | ,487 | ,912 |
| t3_D_Q6_avoiding thinking or talking about it | 40,88 | 199,245 | ,315 | ,915 |
| t3_D_Q7_avoiding activities | 41,33 | 197,884 | ,376 | ,913 |
| t3_D_Q8_trouble remembering | 41,21 | 192,955 | ,494 | ,911 |
| t3_D_Q9_loss interest | 41,38 | 191,462 | ,498 | ,911 |
| t3_D_Q10_feeling distant | 40,79 | 188,694 | ,521 | ,910 |
| t3_D_Q11_feeling numb | 41,17 | 190,493 | ,570 | ,909 |
| t3_D_Q12_future cut short | 41,46 | 185,476 | ,598 | ,908 |
| t3_D_Q13_falling asleep | 40,96 | 185,781 | ,647 | ,906 |
| t3_D_Q14_feeling irritable | 41,13 | 194,549 | ,445 | ,912 |
| t3_D_15_difficulty concentrating | 40,67 | 192,493 | ,526 | ,910 |
| t3_D_Q16_super alert | 41,00 | 180,783 | ,838 | ,901 |
| t3_D_Q17_feeling jumpy | 41,04 | 176,389 | ,751 | ,903 |
|  |  |  |  |  |
|  |  |  |  |  |
| **Skala: ALLE VARIABLEN DASS21** | | | |  |
|  |  |  |  |  |
| **Zusammenfassung der Fallverarbeitung** | | | |  |
|  | | N | % |  |
| Fälle | Gültig | 23 | 95,8 |  |
|  | Ausgeschlossena | 1 | 4,2 |  |
|  | Gesamt | 24 | 100,0 |  |
|  |  |  |  |  |
|  |  |  |  |  |
| **Reliabilitätsstatistiken** | |  |  |  |
| Cronbachs Alpha | Anzahl der Items |  |  |  |
| ,925 | 21 |  |  |  |
|  |  |  |  |  |
|  |  |  |  |  |
| **Item-Skala-Statistiken** | | | | |
|  | Skalenmittelwert, wenn Item weggelassen | Skalenvarianz, wenn Item weggelassen | Korrigierte Item-Skala-Korrelation | Cronbachs Alpha, wenn Item weggelassen |
| t3_B_Q1 - Hard to wind down | 23,83 | 185,059 | ,426 | ,924 |
| t3_B_Q2-dryness of my mouth | 23,96 | 179,225 | ,444 | ,925 |
| t3_B_Q3-any positive feeling | 24,22 | 192,360 | ,094 | ,931 |
| t3_B_Q4-breathing difficulty | 24,09 | 181,174 | ,512 | ,923 |
| t3_B_Q5-difficulty to initiate things | 23,43 | 181,439 | ,486 | ,923 |
| t3_B_Q6-over reacts | 23,74 | 175,747 | ,672 | ,920 |
| t3_B_Q7-trembling hands | 24,48 | 175,534 | ,669 | ,920 |
| t3_B_Q8-using nervous energy | 23,43 | 178,621 | ,520 | ,923 |
| t3_B_Q9-worried of panick and make fool of my self | 24,26 | 180,292 | ,541 | ,922 |
| t3_B_Q10-had nothing to look forward | 24,39 | 175,249 | ,729 | ,919 |
| t3_B_Q11-getting agitated | 24,13 | 178,846 | ,613 | ,921 |
| t3_B_Q12-difficult to relax | 23,65 | 179,964 | ,572 | ,922 |
| t3_B_Q13-down hearted and blue | 24,00 | 175,727 | ,735 | ,919 |
| t3_B_Q14-intolerant of what kept me on what I was doing | 23,78 | 184,542 | ,515 | ,923 |
| t3_B_Q15-close to panic | 24,13 | 170,209 | ,810 | ,917 |
| t3_B_Q16-unable of enthusiasm | 24,22 | 179,814 | ,479 | ,924 |
| t3_B_Q17-not worth as a person | 24,26 | 176,292 | ,695 | ,919 |
| t3_B_Q18-rather touchy | 23,78 | 177,451 | ,708 | ,919 |
| t3_B_Q19-aware of heart action | 24,22 | 174,087 | ,729 | ,919 |
| t3_B_Q20-scared without reason | 23,96 | 168,680 | ,825 | ,916 |
| t3_B_Q21-life was meaningless | 24,39 | 180,431 | ,559 | ,922 |
|  |  |  |  |  |
|  |  |  |  |  |

# **Appendix F**

**Short focus group interview (with group 3)**

0:01 – Interviewer (Main Co-Therapist, Translator, Farsi/Dari, DMT student)

So, participants, please give your attention to me.

0:57 - Interviewer

So, what changes during these sessions have you felt in your life? In how you behave or how you do in social living with others? Or maybe something that you think better, your memory is better, okay? Or emotionally? Feelings. Yeah, feelings or emotionally. Very thin. Can you please translate if you understand me? Okay.

2:34 - Speaker 3

In my case, it was a relationship with a man. I became less aggressive. I started to think less about him and pay more attention to myself. And he somehow automatically attracted me. We had a bad relationship for three years. And then it came to a divorce. I wanted to put an end to it. And now he comes and sits with the child. I don't know how it is connected. Maybe because I became more smiling, more cheerful. I have such changes.

3:09 - Speaker 4 (Translator; Ukrainian Co-Therapist, DMT Student)

The relationship with her husband got better. They had some issues for three years. And now she feels like she started taking better care of herself. And that's why he's also reacting differently, that he takes care of her and of the child. And maybe because she became more cheerful. He's taking better care of himself.

3:29 - Interviewer

Thank you.

3:33 - Speaker 1

Yeah, sorry. Yeah. Firstly, I just want to say thank you to everyone for the opportunity to be here, from the instructor, leader, and the organization of this group. Yeah. And to my therapist for me to come here. I really see a lot of changes in me. Firstly, the confidence. Yeah, most times, most times when I walk on the road, I cry. Yeah. And I felt, how will I continue to move again? Like, will I be able to walk for myself, be independent, focus? And now I can stand up and look at the mirror. Before, when I look at the mirror, I just cry. I see myself in another image. Yeah. But now, I feel better, a lot of different. I can read. I can take my time to take a book and read without crying or watching a movie and without thinking about my past or just walking maybe on the streets and something just crossed my mind. I won't be able to concentrate more and yeah. Thank you. Thank you to everyone, to the We, participants, I say thank you to the leaders. Yeah, this, because I never thought something like dance, I was like, what is dance from When I first heard about it, I said, I would like just come here to be dancing and it's not going to change anything in me. So that means I'm wrong, and I really appreciate everything. Thank you. Thank you.

5:13 - Speaker 0

Yeah. What can I say? I'm very, very grateful. As she has already said, I thank the team. I thank the person who came up with this idea because it has gone a long way to help me. And I think some of us, many of us, this is my second time. It has really helped me. Yeah. Um, because I was really down when She made me. Yeah, I was really down. But now, I am fine. I cannot lie. I am fine. And thanks to this therapy. When Thursday is coming like this, I am very happy. The day I don't come, I am not happy. And as it is finishing, I am not happy. If it has to be continuing, continuing, I will be part of it. I would want to be because when I come here, I am fine. When I go back home, I concentrate. I thank whoever gave my name to her until they had to put me inside the group. I am very, very grateful that people are seeing into our worries, into our problems, making us at least mental health is the best thing so far. So I am very, very grateful and I pray that you continue with the work. To see into it that we are fine. At least that's all I have to say. Thank you very much. I'm very, very grateful. It has really changed me. Today I came, I was feeling pains again. Immediately I danced the pain disappeared. I had pain here, it disappeared. In your shoulder? Yeah, because this is something I cannot do alone in the house. But when I'm here, I'm forcing to do it too. So I'm very, very happy. It has helped me a lot. Thank you very much.

7:10 - Speaker 11

Thank you.

7:11 - Interviewer

Concrete change on the body level. Yeah. But you can also say something negative changed, okay? You don't need to stick to the positive.

7:18 - Unidentified Speaker

Negative.

7:20 - Speaker 4

Negative.

7:21 - Speaker 0

I don't have any negative, I see only positivity. And I want it to continue like that, only positivity because from our experiences and from what each and every one of us say, at least it is of great help to us. Yeah, especially me.

7:38 - Speaker 1

The negative is just that I'm far from here.

7:41 - Speaker 7

Yeah.

7:41 - Speaker 1

And despite I'm going to a Deutsch course, I still take my time to come because I'm always looking forward to this day. I don't know. Sometimes before I get home, say 10 p.m. At night, or maybe sometimes because of the train. Yeah, that's just it.

7:59 - Interviewer

Thanks for being so courageous and do all this to come. Yeah.

8:26 - Speaker 3

I couldn't solve this problem for half a year. I decided to go to a dance therapy. It would be good if I didn't have any pain. I invited a person and they fixed everything. It was very easy and without money.

I want to learn dance therapy because I have an art therapy education.

9:15 - Speaker 4

She had her back fixed even before coming to dance therapy and then she said that she has no more desire for aesthetic things like buying flowers, taking care of herself, having things arranged nicely at home.

9:31 - Interviewer

She does this now? Yes.

9:34 - Speaker 4

And she also wants to get an education of dance therapist because she is an art therapist herself.

9:42 – Speaker 10

Okay. I love it.

9:43 - Speaker 3

Thank you. Thanks for sharing. I love you.

9:48 - Interviewer

And anyone else wants to share something? Yes, I want.

10:00 - Speaker 7

Yes, please. AA says thank you to you all for this project and that they found you and became a part of this project.

10:19 - Speaker 7

I understand that this is a colossal work for you, and you have done a lot. I will try to explain what exactly for me, but I see the reaction of all the girls before and after, and during the process, how we feel. And thanks to you, that you made this project and gave the opportunity to be busy every week, to come here. This means a lot in Germany.

10:47 - Speaker 7

We knew that once a week, no matter what happens. When you are busy, don't you think about The fact that you're busy and that you have something you're looking forward to makes you feel in a different way and you do not think about your own problems or issues that you have that you're looking forward to.

11:45 - Speaker 7

Specifically, your project helped me to complete chemotherapy, I just finished you, to undergo surgery and to bury my father. And if it were not for the dental therapy, I would probably have been experiencing it all for a long time, and there would have been triggers in the body, and it would have lasted longer. Here, through the dance, I shone it all out.

12:10 - Speaker 4 (Translator, Ukranian DMT-student)

Personally for A, this project helped her to go through chemotherapy, to go through the operation and her father died as well during this time. And without dance therapy, she says, it would be much more difficult for her to handle it. And here, through the dance, she could express it.

12:30 - Speaker 2 (Turkish Co-Therapist, DMT student))

It helped her body to open up.

12:55 - Speaker 4 (Translator)

And she even landed in a theater project. She was inviting you in the WhatsApp group. And she says that it means that the body is, there is a body work ongoing.

13:08 - Speaker 2 (Turkish Co-Therapist, DMT student)

I'm sorry, she felt safe? She felt safe coming here, naturally. It's a safe environment.

13:24 - Speaker 7

It is worth a lot and it means a lot to her. Thank you for coming, for sharing. Anyone else?

13:51 - Speaker 11

Thank you.

14:27 - Unidentified Speaker

It's difficult to get together.

14:47 - Speaker 11

Thank you. Thank you.

15:23 - Interviewer

So, does anyone want to share anything else?

15:47 - Speaker 4 (Translator)

Every week she was waiting for the next week to come to Dance Therapy and she didn't miss even one time of our group and she enjoyed every single group.

15:58 - Speaker 8

During Dance Therapy I had a lot of changes. I signed up as a Sprachpartner to learn German. My German started to settle in my head a little bit. I additionally work from home and I found a job.

16:15 - Speaker 4 (Translator)

During this time she had also some changes. One of them was she had a new Sprachpartner German course and her German started being more clear for her and she even found a job.

16:29 - Speaker 4 (Translator)

She says when she feels bad at work she starts dancing and she found a nice work place for her because she's a waitress at the place where there are lots of weddings. So it's atmosphere and a chance to dance.

17:01 - Speaker 8

With the help of dancing and a good mood, I go around the whole island.

17:05 - Speaker 8

I'm a mom. I also did the general cleaning at home, threw out old things. I have a rearrangement, flowers on the windowsill, a beautifully decorated window.

17:33 - Speaker 4 (Translator)

She also made a lot of changes at her place where she lives. She cleaned up, threw away the old stuff, put some flowers or plants on the window, so she made the rearrangement.

17:44 - Speaker 8

I had very few classes, I would like to repeat this course, because I have a very serious block and I do not experience any emotions at all. And I have two more friends, two dogs.

17:59 - Speaker 8

She said two things. One thing was that eight times was not enough for her. She would like to get more, especially because she has an emotional block and she doesn't feel emotions.

18:24 - Speaker 4

And the second thing, she said that she wanted to start practicing German to her landlords, with her landlords. But then she ended up becoming friends with their dogs. And every time she hugs them or plays with them, they're licking her. So she's doing that, too. And now she likes dogs before she was a cat person.

18:50 - Interviewer

Oh, OK. Thank you. Wish you luck.

19:01 - Interviewer

Yes, please.

19:04 - Speaker 5

As you know, I came here with suicidal thoughts and I was really, it was terrible time. I was on the medication and stuff. And when the therapy started, actually I stopped taking medication and I was afraid of this effect.

19:19 - Speaker 5

When you stop it, And it really helped me to go through this period, even without tablets. And I can't say that something drastic has changed. However, I don't want to kill myself anymore. And even though I had a couple emotional breakdowns during the sessions, but it was really deep. And I think it was also good because As someone mentioned, I felt safe here to actually show these emotions and live through them whatever was happening around. So thank you very much for everybody in the group for this feeling of safety. It was really cool, so thank you for that. Thank you. And I also have this crazy idea of considering dance therapy as my another education, because I've always been into psychology, into therapy. Yeah, why crazy then? Here in Germany, everything seems to be crazy when you are a refugee, I mean.

20:26 - Interviewer

You can if you want to. Thank you.

20:46 - Speaker 5

Okay, I'll just briefly say that I came here with suicidal thoughts. I was at the psychiatrist on pills. And just when the therapy started, my period was already over, and I was worried that I would be in a coma. And with this therapy, everything went well for me, I'm okay. I can't say that I had some super changes in my life, but at least I don't want to end my life.

I had some emotional breakdowns here, including during the semi-session, but the emotions had to go. And I am very grateful to all the groups for the fact that I felt safe to do this. So thank you very much.

I had a crazy idea to become a dance therapist. I've always wanted to be a psychologist and a therapist. I have a chaos. Everything can be perfect, but I had a request. To find myself, to come to myself. And in this way, I lost everything. I have nothing. Nothing loved. I was left alone. I understand that I need to start from scratch.

22:37 - Speaker 4 (Translator)

O is feeling very chaotic or in general a chaotic state in her life. She said that when she came here she had a need to find herself, to come the way to herself. And currently she ended up being alone and she lost her beloved man. But she understands that she needs to start from zero, from scratch. But dancing has always supported me since childhood.

23:09 - Speaker 11

I danced and love for children, so to speak, has always kept me in tune. And I understand how important it is. Dancing helps a lot. If I knew the language, I would also go to school, of course. But I have chaos in my head, chaos in my room. I can't focus only on the language to teach it. A lot of pain has risen. Disappointment. Well, in short, all that. I realize a lot of things. I realize that I was not valuable for myself. And a lot of such things.

23:59 - Speaker 12

She said that dance supported her from the time when she was a child and supports her now and she says that a lot of things from her subconscious come out and she realizes a lot of things about herself. But

24:30 - Speaker 4 (Translator, Ukrainian DMT student)

she feels very chaotic still, on the outside and on the inside, also at her room or at her apartment. And if she knew the language, she would also continue learning about dance therapy. But at the moment she cannot concentrate on the language.

24:49 - Interviewer

Do you think these sessions can help you? In what way can help her to go through the stuff that she's going through now?

25:12 - Speaker 12

She says it's a huge support from the group and from those who do the group. And she can be herself, she can express herself, she can dance, she can scream, she can be herself and not be judged.

26:04 - Unidentified Speaker

I would say that I was more physically and socially active because I don't socialize at all. I like to be with myself all the time. So it was something different and I would especially thank K (The therapist) for doing these sessions unpredictable and trying different things. Thank you so much. That's all. Thank you.

26:52 - Unidentified Speaker

Okay, anything else anyone wants to add?

26:57 - Unidentified Speaker

Thank you.
